# Multiple stressors in multiple species: Effects of different RDX soil concentrations and differential water-resourcing on RDX fate, plant health, and plant survival

**DOI:** 10.1371/journal.pone.0234166

**Published:** 2020-08-14

**Authors:** Richard F. Lance, Afrachanna D. Butler, Carina M. Jung, Denise L. Lindsay

**Affiliations:** Environmental Laboratory, United States Army Engineer Research and Development Center, Vicksburg, Mississippi, United States of America; National University of Kaohsiung, TAIWAN

## Abstract

Response to simultaneous stressors is an important facet of plant ecology and land management. In a greenhouse trial, we studied how eight plant species responded to single and combined effects of three soil concentrations of the phytotoxic munitions constituent RDX and two levels of water-resourcing. In an outdoor trial, we studied the effects of high RDX soil concentration and two levels of water-resourcing in three plant species. Multiple endpoints related to RDX fate, plant health, and plant survival were evaluated in both trials. Starting RDX concentration was the most frequent factor influencing all endpoints. Water-resourcing also had significant impacts, but in fewer cases. For most endpoints, significant interaction effects between RDX concentration and water-resourcing were observed for some species and treatments. Main and interaction effects were typically variable (significant in one treatment, but not in another; associated with increasing endpoint values for one treatment and/or with decreasing endpoint values in another). This complexity has implications for understanding how RDX and water-availability combine to impact plants, as well as for applications like phytoremediation. As an additional product of these greenhouse and outdoor trials, three plants native or naturalized within the southeastern United States were identified as promising species for further study as in situ phytoremediation resources. *Plumbago auriculata* exhibited relatively strong and markedly consistent among-treatment mean proportional reductions in soil RDX concentrations (112% and 2.5% of the means of corresponding values observed within other species). Likewise, across all treatments, *Salvia coccinea* exhibited distinctively low variance in mean leaf chlorophyll content index levels (6.5% of the means of corresponding values observed within other species). Both species also exhibited mean wilting and chlorosis levels that were 66% and 35%, and 67% and 84%, of corresponding values observed in all other plants, respectively. *Ruellia caroliniensis* exhibited at least 43% higher mean survival across all treatments than any other test species in outdoor trials, despite exhibiting similar RDX uptake and bioconcentration levels.

## Introduction

One of the unique challenges to plant health on military ranges is environmental contamination with unique compounds required for military training and operations. One such compound is the nitroaromatic Royal Demolition Explosive (RDX; hexahydro-1,3,5-trinitro-1,3,5-triazine). Unignited RDX can leach into soil and groundwater and is a relatively common contaminant on military firing ranges [1, 2]. Historically, RDX contamination of soils and waters also resulted from inadequate waste disposal practices at chemical and munitions production facilities [3, 4]. RDX and its associated metabolites are absorbed by plant roots, then transported to stems, leaves, and flowers [5]. The majority of RDX may remain untransformed and accumulate in leaf tissues [3, 6, 7].

RDX soil contamination can be associated with negative trends in plant growth, survival, and health indicators, such as declines in chlorophyll concentrations or increased leaf chlorosis, necrosis, and/or curling [2, 7–10]. However, RDX can affect different species differently [10–12]. In a study of RDX impacts on 18 terrestrial plants [13], 16 species exhibited reduced growth and two exhibited enhanced growth at various RDX soil concentrations. Via et al. [14] noted that basic knowledge regarding the effects of RDX on wild plants and plant communities is lacking, including how RDX impacts different plant life stages. According to the same authors, additional research is needed to better understand how naturally occurring plant species respond to explosive contamination, and to better clarify the mechanisms involved in such responses.

In a period where natural resource managers, conservation professionals, and environmental planners are increasingly concerned about broad shifts in climate and changes in the frequencies of interannual climate extremes [15–18], interactions between climate and additional, other stressors are of particular interest. For example, air pollution can impair tree responses to freezing stress and drought conditions [19], and ultraviolet radiation can increase soil contaminant phytotoxicity [20]. Water availability (e.g., annual precipitation) is one of several climatic factors for which both long-term shifts and short-term lability are of particular concern [21–23]. Water availability, whether in excess or deficiency, can stress plants, and can influence chemical phytotoxicity [24]. It also has the potential to influence the effects of RDX on plants, as transpiration significantly influences the movement of minerals, nutrients, and contaminants throughout a plant [25]. While interactions between climatic factors and RDX soil contamination are not well known, one study [8] found no interaction effect between water deprivation and RDX soil contamination on the production of anthocyanin and changes in leaf coloration in *Sida spinosa* L. (Malvaceae). Additional studies of a similar focus appear to be lacking, with more research needed. We report on the combined effects of different RDX soil concentration (50 ppm and 100 ppm) and different water-resourcing levels (where water-resourcing refers to the amount of water provided to plants) to plant survival and health (leaf wilting, chlorosis, and chlorophyll content) for a total of nine different plant species. Impacts to leaf vitality, structure, and appearance are among the most common manifestations of RDX phytotoxicity [26]. Limited RDX bioaccumulation data for each species under different treatments are also provided. This is done primarily to demonstrate RDX uptake and accumulation by plants, though some informative comparisons among treatments was also possible. The soil RDX concentrations used for these trials are well within those reported for RDX concentrations on contaminated sites [27].

In addition to exploring how a second stressor (water availability) impacts plants, particularly in the case of munitions compounds, our study also has the potential to identify plants that may be of further interest in terms of phytoremediation. Phytoremediation is one of several measures available for removing RDX and other military-associated contaminants from soils [28], and can be defined as a “process that uses plants, green vegetation, trees, aquatic plants, and grasses, to remove, stabilize, transfer, and/or destroy toxic pollutants from surface water, groundwater, wastewater, sediments, soils, and/or external atmosphere” [29]. We were particularly interested in in situ phytoremediation (i.e., occurring in place on contaminated soils), and in the use of native species as potential phytoremediation resources. Native plants have potential advantages over non-native species for in situ phytoremediation in terms of adaptations to local conditions (e.g., climate, mycorrhizal communities, herbivorous insect pests), insertion within local ecological networks and community dynamics (e.g., pollination), and reduced risk of becoming problematic invasive species [30, 31]. With that secondary objective in mind, we included three species native to the southeastern United States (*Conradina canescens*, *Ruellia caroliniensis*, and *Salvia coccinea*) in our trials.

## Materials and methods

### Greenhouse trial

#### Plant establishment and experimental treatments

For each of eight plant species (Table 1), 24–36 individual plants were potted in “clean” or RDX-infused soils and maintained in a greenhouse located on the US Army Engineer Research and Development Center’s (ERDC) Waterways Experiment Station (WES) in Vicksburg, MS. Soils were prepared using dry RDX (1% HMX; BAE System, Ordnance Systems Inc., Holston Army Ammunition Plant, Kingsport, TN) that was ground with a mortar and pestle into a fine powder and dissolved in acetone, then mixed to target concentrations of 50 ppm and 100 ppm in a 10:3 silica quartz sand:loess soil (loess local to study area). Both the silica quartz sand and loess soil were both considered “clean”, as no background RDX was detected during analysis. Plants were removed from their original containers, roots moistened with municipal tap water, and then transplanted in clean or RDX-infused soils in 1-gallon (16.51 cm in diameter by 15.88 cm in depth) and 2-gallon (22.86 cm in diameter by 21.59 cm in depth) pots, respectively, depending on plant size. Approximately 3 and 8 kg of each of the clean or RDX-infused soils were added to the 1- and 2-gallon pots, respectively.

**Table 1:** Plant species and sample sizes per treatment (*n*_t_) for greenhouse studies.

Species	Common Name	*n*_t_	Source
*Antirrhinum majus*	Snapdragon	5	Local home and garden store
*Dianthus*	Pink	5	Local home and garden store
*Hibiscus mocheutos*	Rose mallow	5	Local home and garden store
*Pentas lanceolata*	Starcluster	5	Local home and garden store
*Plumbago auriculata*	Cape leadwort	2–3	Local home and garden store
*Ruellia caroliniensis*	Wild petunia	4	Regional native plant nursery
*Salvia coccinea*	Scarlet Sage	5	Regional native plant nursery
*Tulbaghia violacea*	Society garlic	2–4	Local home and garden store

Plants (n = 3–5) in each of the 0, 50, and 100 ppm soil concentration groups were further assigned to either a 1X or 0.5X water-resourcing treatment (Table 2), which corresponded, roughly, to receiving, every other day, 1 L (1X) or 0.5 L (0.5X) of municipal tap water (City of Vicksburg; sourced from groundwater wells drawing from the Mississippi River Alluvial Aquifer). We assumed that at least one of the two levels of water-resourcing (1X or 0.5X) would constitute a stressor for each species, being comparatively further from the optimal water-resourcing level for that species under greenhouse conditions. Miracle-Gro^®^ Water Soluble All Purpose Plant Food (Scotts Miracle-Gro, Marysville, OH, USA) was included at an approximately 1:250 dilution with watering once per week. Plants were maintained under treatment conditions (Table 2) for approximately 19 weeks (133 days).

**Table 2:** Water-resource and RDX soil concentration treatment classes employed in greenhouse trial.

Water Resourcing Level | RDX Soil Concentration Level		
0.5X | 0 ppm	0.5X | 50 ppm	0.5X | 100 ppm
1X | 0 ppm	1X | 50 ppm	1X | 100 ppm

#### Measuring RDX concentrations in soil and plant tissues

RDX soil concentrations were measured at the start and end of trials, and in root tissues at trial completion. In all cases, RDX was extracted and measured using methods specified in EPA SW-846 Method 8330 [32]. Because of limited availability of above ground tissues, RDX concentration data for these tissues were not included in later analyses (though limited data for leaf and flower tissues are provided in S1 File and S1 Table). Proportional reductions in soil RDX concentration (*PRC*s) for each unit (plant x species x treatment) were calculated by dividing the final soil RDX concentration in each plant (S2 Table) by the overall mean soil RDX concentration for the treatment group (species x treatment), and then subtracting that value from 1. Bioconcentration factors (*BCF*s) for RDX in root tissues were calculated by dividing RDX concentrations in root tissue (S2 Table) by the initial RDX concentrations in the soil.

#### Determining plant health endpoints

Upon approximately four weeks of growth under treatment conditions, three plant health endpoints (or stress metrics) were measured, namely the level of wilting (*W*) visually observed on leaves, the level of chlorosis (*C*) visually observed on leaves, and a chlorophyll content index (*CCI*; complete data found in S3 Table). Wilting, or areas of dead tissue, was visually estimated for four randomly-selected leaves per plant. Each leaf was placed into one of four classes, based on estimated extent of wilted leaf surface area: 0–25%, 26–50%, 51–75%, 76–100%. A mean wilt level for each plant was then calculated, which was then used to calculate a mean wilting level for each treatment ($\bar{W}$) within each species. Observations on chlorosis, defined as areas of notably reduced or absent green hue in leaf tissues, were made, as well, and a mean leaf chlorosis ($\bar{C}$) for each treatment group in each species was determined in the same fashion as for wilting Additionally, for each selected leaf, one to three *CCI* measurements were made (depending on leaf surface area) using a CCM-200 plus chlorophyll content meter (Opti-Sciences, Inc.; Hudson, NH, USA). Either a single *CCI* value or mean leaf *CCI* was recorded for each leaf and mean *CCI* was calculated for each plant, and a mean CCI ($\bar{CCI}$) for each treatment group in each species. The CCM-200 Plus uses two LED sources to measure light transmittance at two wavelengths: 931 nm, which falls within the chlorophyll absorbance range, and 653 nm, which provides a weighting factor associated with mechanical differences such as tissue thickness. *CCI* is the product of percent light transmittance at 931 nm and the inverse of the percent light transmittance at 653 nm. While not equivalent to actual density of chlorophyll in plant tissues, *CCI* provides a useful metric for comparing chlorophyll content among different samples.

### Outdoor plot trial

#### Outdoor plot establishment and experimental treatments

We selected three plant species, *P*. *lanceolata*, *R*. *caroliniensis*, and *Conradina canescens* for outdoor trials with single and combined water-level resourcing and RDX soil contamination treatments (Table 3). The former two species were included in this trial because they had provided a relatively consistent and productive bloom set in the greenhouse, and the outdoor trials were also part of a concurrent pollination study. The latter species, *C*. *canescens*, had been observed to thrive on the outdoor plot in an earlier, unpublished pilot study. Individual plants of this species were obtained from a regional native plant nursery and included in this trial, despite being excluded from the earlier greenhouse trials due to rapid mortality across all treatments.

**Table 3:** Water-resource and RDX soil concentration treatment classes employed in outdoor plot trial.

Water Resourcing Level | RDX Soil Concentration Level	
0.5X | 0 ppm	0.5X | 100 ppm
1X | 0 ppm	1X | 100 ppm

**Fig 1:**
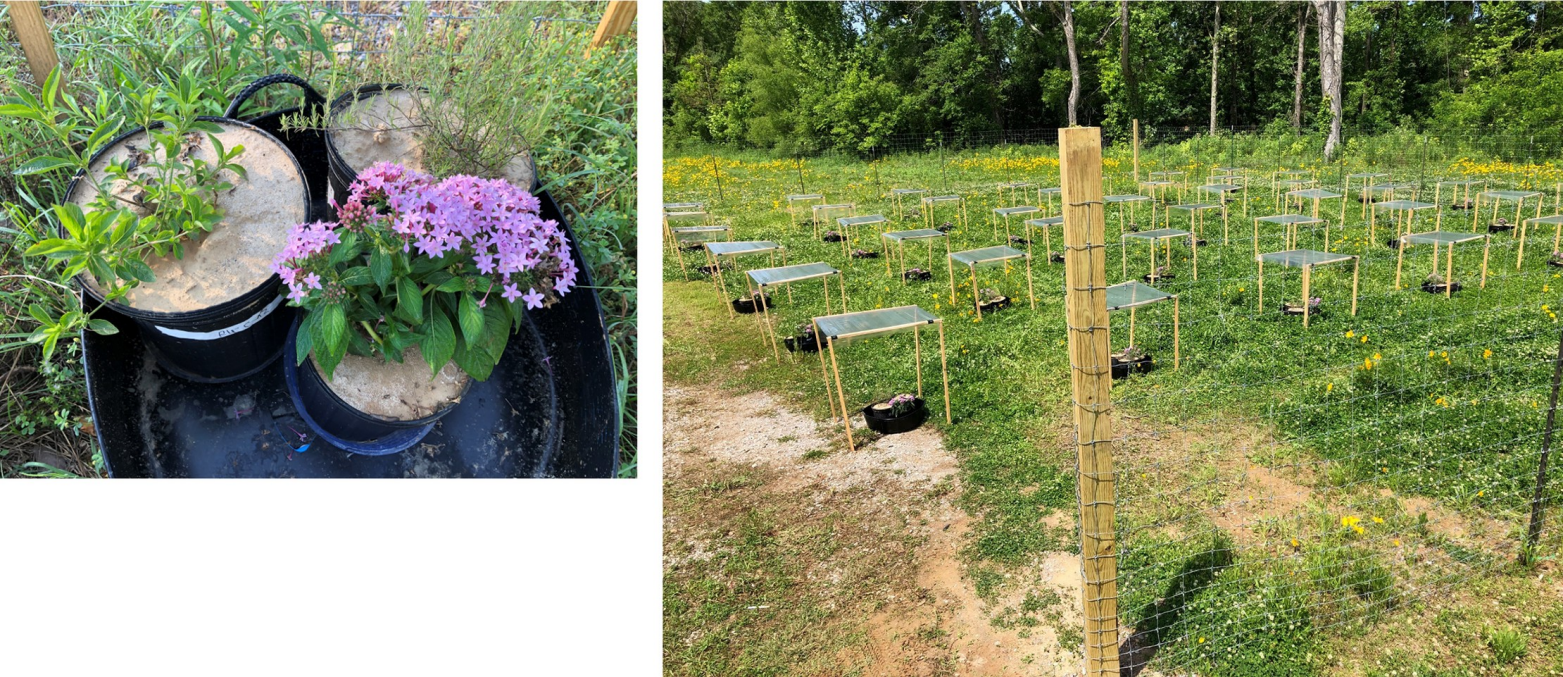
Images of outdoor plot at commencement of trial. *Above*, outdoor plot station with one potted plant per species for *R*. *caroliniensis* (left), *C*. *canescens* (upper right), and *P*. *lanceolata* (lower right). *Right*, outdoor experimental grid of 48 stations.

Individual adult plants (n = 48 per species) were repotted into sandy loess soil containing either no RDX (control) or 100 ppm RDX (prepared using methods described above). A section of a grassy field at ERDC-WES was developed as an outdoor study plot. The plot area was fenced to exclude large herbivores. Within the study plot, one individual potted plant from each species was placed into a 35 L black rubber basin which had been placed on a plot “station” (Fig 1). Each station was centered underneath a 0.9 m high clear polyethylene rain shield. A total of 48 stations were positioned in a grid with columns alternated between plants in control and RDX-contaminated soil. The first four rows of the grid was assigned to a 1X water-level treatment (approximately 1 L) while the second set of four rows was assigned to the 0.5X treatment (approximately 0.5 L). All plants were watered two to three times weekly, depending on weather. The plots were inspected for dead plants once per week for 10 weeks. After the end of this period, soil and shoot/leaf tissue were collected for measurement of RDX concentrations and calculation of *PRC*s and *BCF*s (as described previously; complete soil and shoot RDX concentration data available in S4 Table).

### Statistical analysis

For all analyses, because of relatively small sample sizes (n = 2–5), we set the critical value for assigning statistical significance at α = 0.10. The majority of treatment response datasets for all endpoints did not meet assumptions for parametric tests, so appropriate nonparametric tests were used. The effects of the main factors (water-resourcing levels, RDX soil concentration) and an interaction factor on each endpoint in the greenhouse trial (*PRC*, *BCF*, *W*, *C*, *CCI*) were tested using robust analysis of variance (RAOV [33, 34]), provided in the package Rfit (version 0.24.2; [35]) in R (version 3.6.1 [36]). For each endpoint, pairwise comparisons of different treatment groups within each species were conducted using Dunn’s test [37], with a Bonferroni multiple comparisons correction to the critical value. Dunn’s tests were conducted using the R package FSA (version 0.8.25 [38]). The effect directions for significant factors in the various tests were assessed using interaction plots based on median values for each endpoint and rank-based estimates of regression coefficients [35].

For the outdoor plot trials, differences in *PRC* and *BCF* between plants of each species in the 0.5X|100 ppm and 1X|100 ppm treatment groups were assessed using one-sample Wilcoxon tests, performed in base R. For each species in the outdoor plot trial, differences in survival (*S*) among treatments with different levels of the main factors or among plants in the different combined treatments (Table 3) within each species were assessed at each time point (plot inspection dates) using log-linear regression analysis, as calculated using the loglm function in the R package MASS (version 7.3–51.4 [39]). Pairwise comparisons of plant survival under different levels of each of the two main factors, or among the different combined treatments were assessed using chi-square tests with Bonferroni adjustments to critical values for multiple comparisons.

## Results and discussion

This study explored patterns in plant responses to an important military soil contaminant, the phytotoxic compound RDX, in the presence of an additional factor (and potential stressor), water-resourcing levels. We conducted tests with a total of nine plant species, none of which had previously been tested for responses to RDX soil contamination.

### Greenhouse trials: RDX uptake and plant health endpoint indices

**Fig 2:**
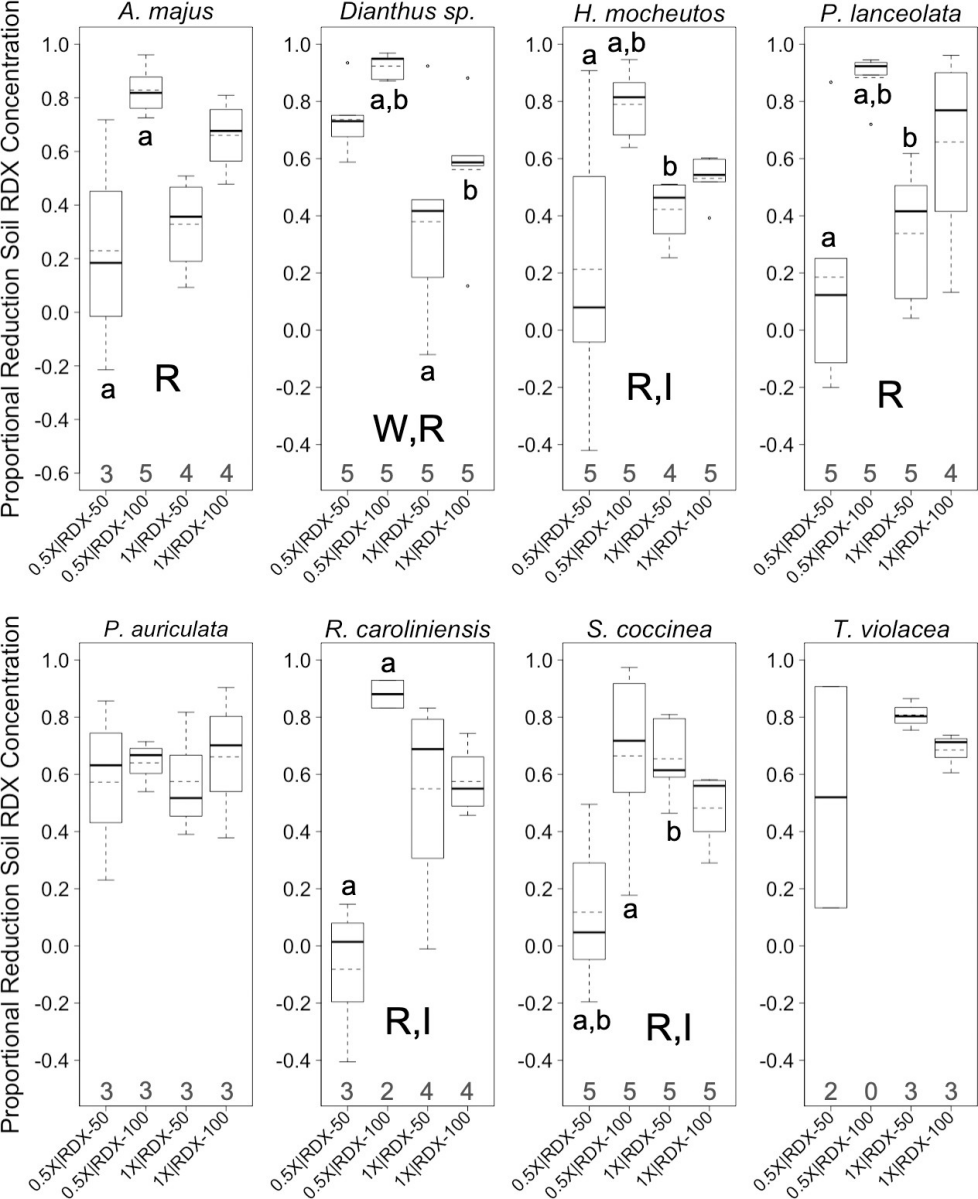
Proportional reductions in soil RDX in greenhouse trials. Boxplots of proportional reductions in *soil* RDX concentrations (*PRC*s) for eight plant species reared for 133 days in a greenhouse under different combinations of two factors, water-resourcing level (1X and 0.5X) and soil RDX concentration (50 and 100 ppm). Boxplots include median (bold horizontal line) and mean (dotted horizontal line) values. Sample sizes within each group are found on the x-axis. R = significant effect of soil RDX concentration; W = significant effect of water-resourcing level; I = significant interaction effect; a, b = significant pairwise difference between treatment groups. α ≤ 0.10.

**Fig 3:**
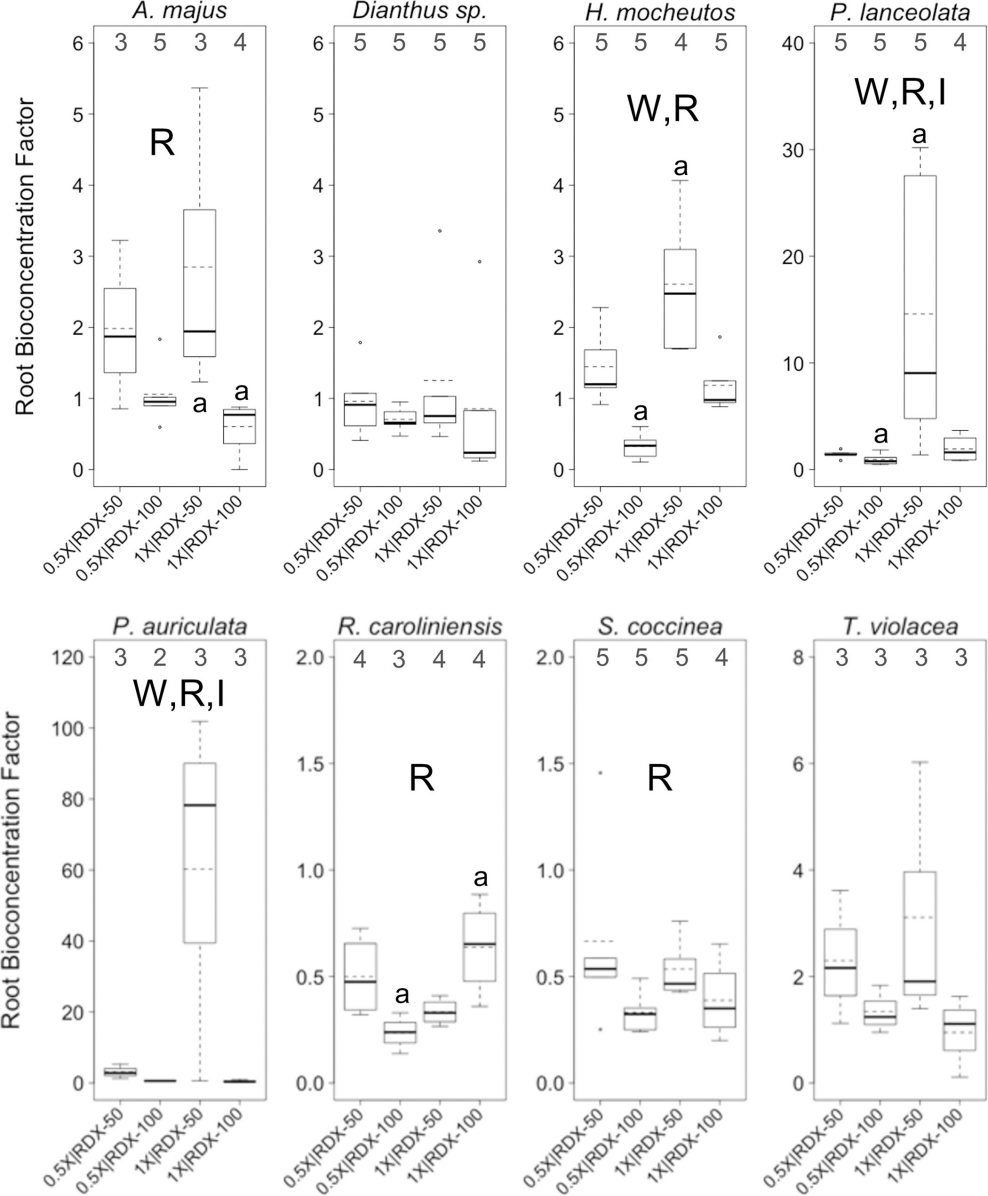
RDX bioconcentration factors in greenhouse trials. Boxplots of final *root* RDX bioconcentrations factors (*BCF*s) for eight plant species reared for 133 days in a greenhouse under different combinations of two factors, water-resourcing level (1X and 0.5X) and soil RDX concentration (50 and 100 ppm). Boxplots include median (bold horizontal line) and mean (dotted horizontal line) values. Sample sizes within each group are found along the top of each boxplot frame. R = significant effect of soil RDX concentration; W = significant effect of water-resourcing level; I = significant interaction effect; I = significant interaction effect; a = significant pairwise difference between treatment groups. α ≤ 0.10.

We were able to maintain all eight plants species (Table 1) in all treatments in the greenhouse for the entire study period. However, because the 0.5X | 100 ppm treatment group samples for *T*. *violacea* were spilled during sample processing and after remaining pot soils had been discarded, *PRC* could not be calculated for this treatment group in this species. Final soil *PRC*s (proportional reductions in RDX soil concentration) and root *BCF*s (bioconcentration factors) varied widely among the eight plant species and treatment groups (Figs 2 and 3). In a few cases, soil samples had negative *PRC*s (and thus measured RDX concentrations above starting concentrations; Fig 2). The initial soil RDX concentrations measured at the point of establishing plants in pots (time point 0) had *mean* RDX concentrations of 50 ppm and 100 ppm (data not shown), and individual measures exceeding these mean values at the end of the trial likely represent, to some degree, nonuniform mixing of RDX in soil and associated random sampling error.

RDX soil concentrations were a significant factor in *PRC* and root *BCF* for a majority of species (Figs 2, 3). In many cases, *PRC* values for the 100 ppm RDX treatments were higher than with the 50 ppm RDX treatments, but pairwise comparisons were not always statistically significant (Fig 2). In terms of bioaccumulation, the 50 ppm RDX treatments generally exhibited higher root *BCF* values than treatments with 100 ppm RDX, though, again, pairwise comparisons were not always statistically significant (Fig 3). As a single factor, water-resourcing had little effect on *PRC* across species and treatments (Fig 2), but was more commonly a factor in root *BCF* (Fig 3). In three plant species, reduced water-resourcing was associated with lower root *BCF*s (Fig 3). Of particular interest, significant interaction effects between the main factors (RDX soil concentrations and water-resourcing levels) were apparent for both *PRC*s and *BCF*s within several species. The 0.5X | 100 ppm treatment resulted in the highest levels of *PRC* for most species, though *PRC*s for this treatment group was not always statistically different from other treatment groups (Fig 2). In terms of root RDX accumulation, the 01X | 100 ppm treatment group often exhibited the highest *BCF*s (Fig 3). Significant interaction effects were observed for both *PRC*s and *BCF*S, in three and two species, respectively (Figs 2 and 3). The strength (significant or not significant) and direction (increasing or decreasing endpoint values) of those effects varied from case to case (Table 4).

**Table 4:** Summary of patterns in RDX fate and plant health in greenhouse trial.

Plant	Soil RDX Reduction (*PRC*)			Root RDX Accumulation (*BCF*)			Wilting			Chlorosis			*CCI*		
	RDX-100^a^	0.5X Water^b^	Inter-action	RDX-100^a^	0.5X Water^b^	Inter-action	RDX-50, RDX-100^C^	0.5X Water^b^	Inter-action	RDX-50, RDX-100^C^	0.5X Water^b^	Inter-action	RDX-50, RDX-100^C^	0.5X Water^b^	Inter-action
*A*. *majus*	↑			**↓**											
*Dianthus sp*.	↑	↑					↑-			↑-			↧↥		
*H*. *mocheutos*	↑		↥	**↓**	**↓**		↕↑		↕	↥-					
*P*. *lanceolata*	↑			↧	↧	↧	↕↕	↕	↕						
*P*. *auriculata*				↧	↧	↧	↑**↓**			↑↑				**↑**	
*R*. *caroliniensis*	↥		↕	↥			↑-	↥	↧	-↥		↥			
*S*. *coccinea*	↥		↕	**↓**			↑-		↕	↑↑					
*T*. *violacea*							↥-	↑				↕			

**Fig 4:**
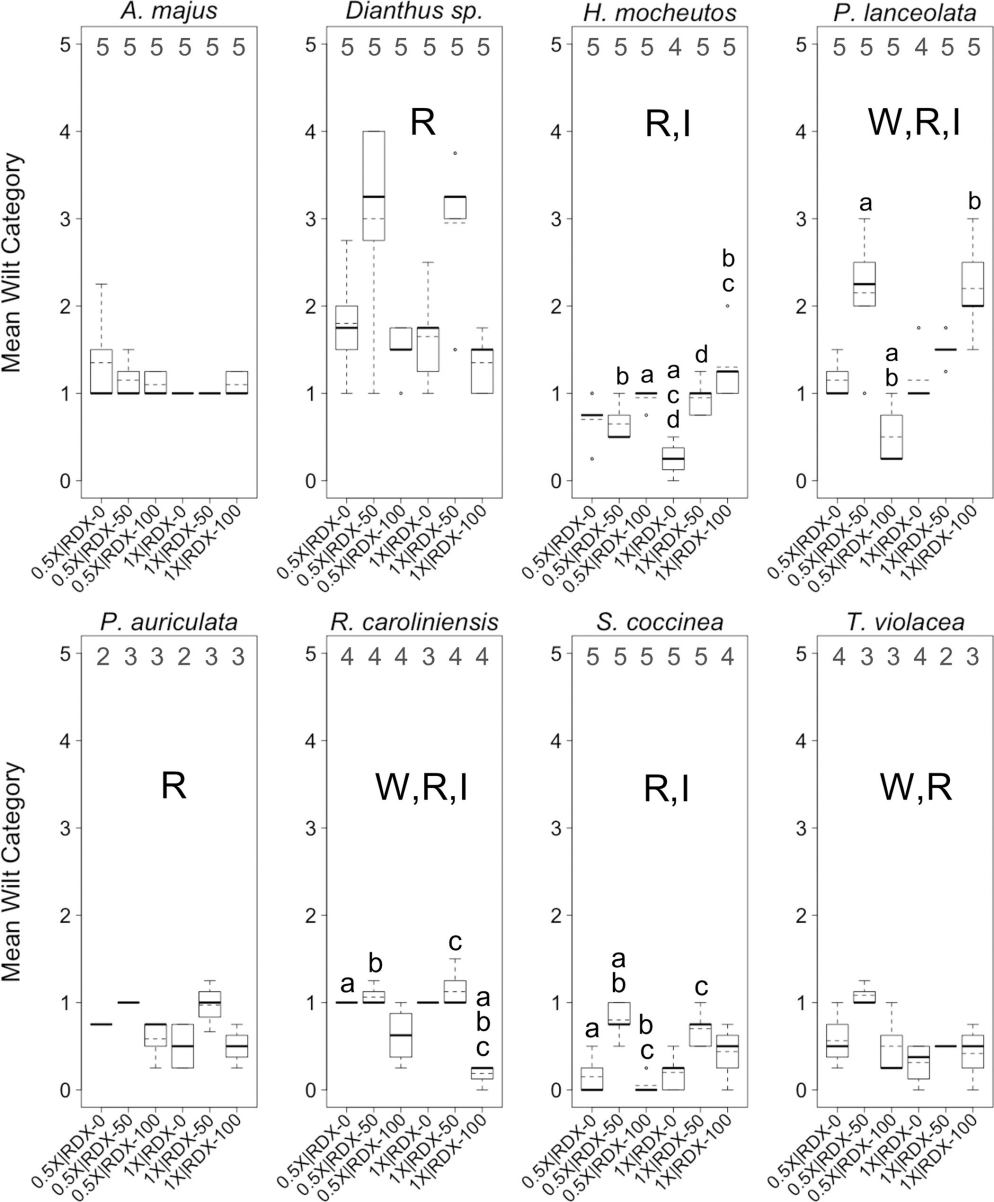
Wilting levels in greenhouse trials. Boxplots of mean *wilting* levels per plant across eight plant species. Plants were reared in a greenhouse under different combinations of two factors, water-resourcing level (1X and 0.5X) and soil RDX concentration (0, 50, and 100 ppm). Boxplots include median (bold horizontal line) and mean (dotted horizontal line) values. Sample sizes within each group are found along the top of each boxplot frame. R = significant effect of soil RDX concentration; W = significant effect of water-resourcing level; I = significant interaction effect; a–d = significant pairwise difference between treatment groups. α ≤ 0.10.

**Fig 5:**
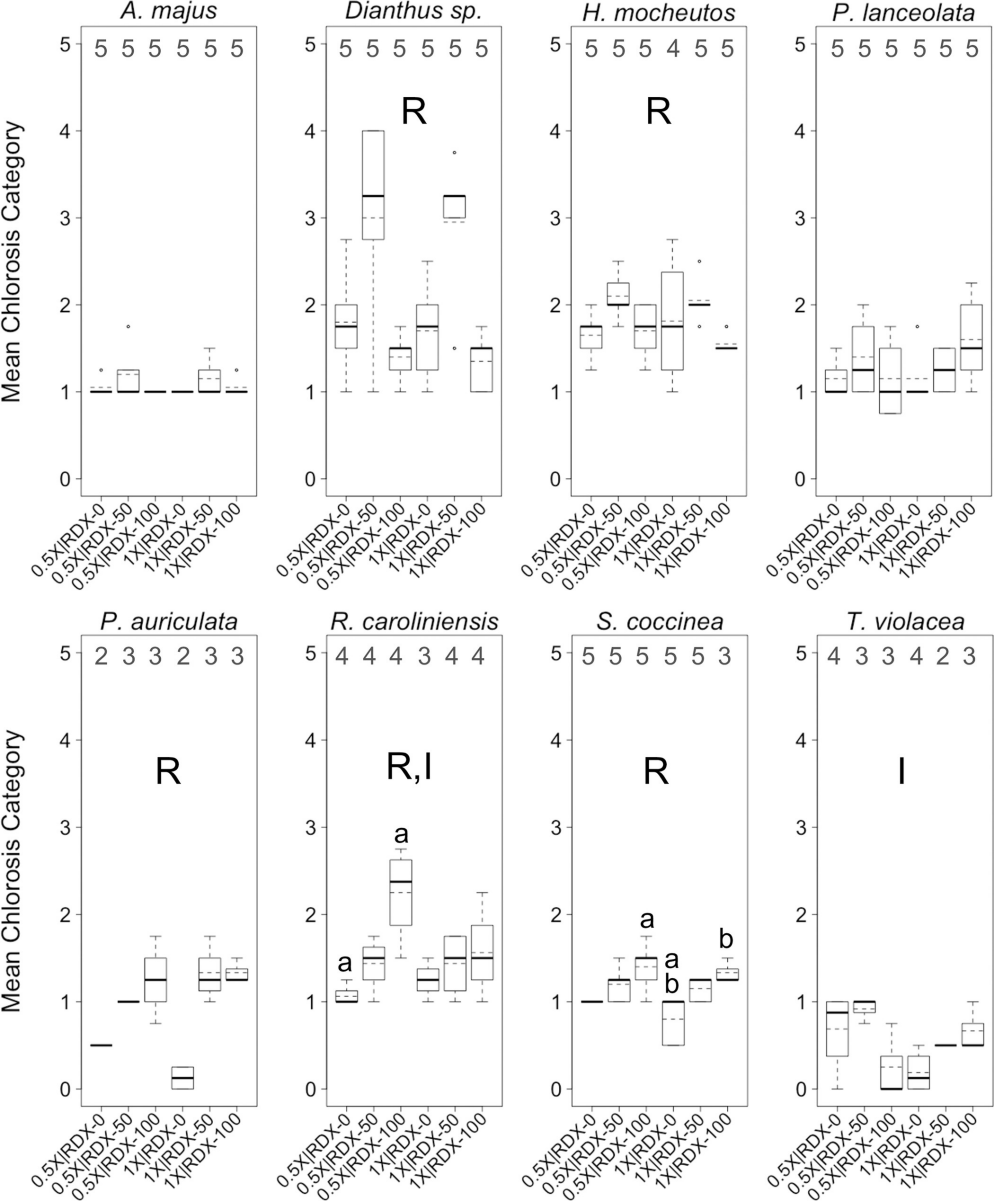
Chlorosis levels in greenhouse trials. Boxplots of mean *chlorosis* levels per plant across eight plant species. Plants were reared in a greenhouse under different combinations of two factors, water-resourcing level (1X and 0.5X) and soil RDX concentration (0, 50, and 100 ppm). Boxplots include median (bold horizontal line) and mean (dotted horizontal line) values. Sample sizes within each group are found along the top of each boxplot frame. R = significant effect of soil RDX concentration; I = significant interaction effect; a–d = significant pairwise difference between treatment groups. α ≤ 0.10.

**Fig 6:**
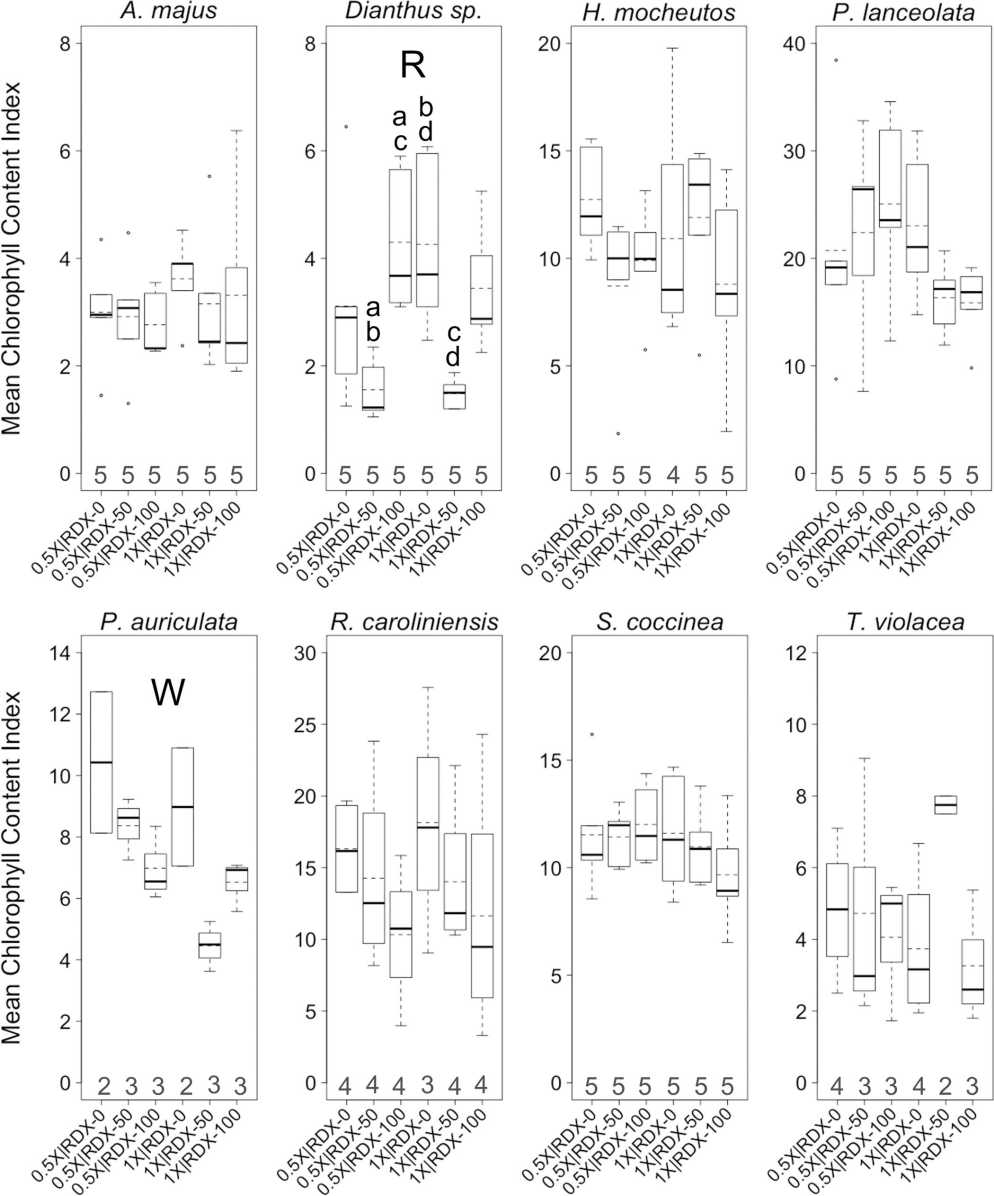
Chlorophyll content index levels in greenhouse trials. Boxplots of mean *chlorophyll content index* (*CCI*) levels per plant across eight plant species. Plants were reared in a greenhouse under different combinations of two factors, water-resourcing level (1X and 0.5X) and soil RDX concentration (0, 50, and 100 ppm). Boxplots include median (bold horizontal line) and mean (dotted horizontal line) values. Sample sizes within each group are found on the x-axis. R = significant effect of soil RDX concentration; W = significant effect of water-resourcing level; a–d = significant pairwise difference between treatment groups. α ≤ 0.10.

Patterns of soil RDX reduction (proportional reduction concentration; *PRC*), root RDX accumulation (bioconcentration factor; *BCF*;), and plant health metrics including leaf wilting, leaf chlorosis, and leaf chlorophyll content (*CCI*) across multiple plant species exposed to different combinations of two main factors (50 ppm and 100 ppm soil RDX concentration, 0.5X water-resourcing level, and interaction effects). Effect directions for factors are provided for those cases in which at least one factor, and in some cases, different treatments, were found to be associated with significant differences in endpoint levels (Figs 2–6). Blank cells indicate that no statistically significant effect was observed. Indicators include ↑ (increase in endpoint value or metric), ↓ (decrease), ↕ (increase and decrease), ↥ (increase and little to no effect depending on treatment groups), ↧ (decrease and little to no effect depending on treatment groups), and–(no discernible effect on a metric).

^a^Relative to effect observed for 500 ppm RDX treatment.

^b^Relative to effect observed with 1X water-resourcing treatment.

^c^Relative to endpoint values observed in treatments without RDX.

In plants, the majority of RDX that is taken up from soil or water media is stored in the above ground tissues, with particularly high concentrations in leaves, seeds, and stems [40]. Thus, it is rather unfortunate that we were unable to obtain a robust dataset of RDX concentrations for multiple tissue-types in our greenhouse trials. However, our primary rationale for making measurements on RDX concentrations in plant tissues was to demonstrate RDX uptake, in support of RDX as a stressor for plants in combination with water-resourcing treatments. The data we provide regarding RDX concentrations in plant roots, and reductions in soil RDX concentrations achieve this primary purpose. We also note that in addition to interspecific variance in *BCF* for RDX in plants, and variance arising from different levels of water resourcing or soil concentrations of RDX, there can be significant variance in *BCF* among different soil types [40]. Such variances hinder extrapolation of *BCF* measures from one scenario or study to another. However, we did observe a general trend of decreasing root *BCF* with increasing soil RDX concentrations across plant species in our study, which is a phenomenon also observable in past studies of RDX uptake and fate in the perennial ryegrass (*Lolium perenne*) [41, 42].

The effects of different water-resourcing levels, soil RDX concentrations, and the interaction of these factors on wilting levels were varied and complex for nearly all of the eight plant species within the greenhouse trials (Fig 4). One general trend was that different RDX concentrations were the most common factor influencing plant health responses to different treatments (Figs 4–6; Table 4). Most plants across treatments and species exhibited only mild wilt (i.e., 0–25%), or in several cases, moderate wilt (i.e., 25–50%; Fig 3). Higher degrees of wilt (> 50% or greater of leaf surface) were relatively rare, and were observed in only a few treatment groups within *Dianthus* and *P*. *lanceolata*. In seven of eight species, there was a statistically significant association between starting RDX soil concentrations and observed differences in mean wilting category. In some cases, higher RDX concentrations appeared to be associated with increasing levels of wilt, yet conversely, in some cases, higher soil RDX concentrations appeared to benefit plants (reduced levels of wilt; Fig 4; Table 4). Differences in water-resourcing levels were associated with significant differences in mean wilting category in three species, with varied effect strengths and directions (Fig 4; Table 4). Significant interaction effects between the main factors were observed in four species, with the strength and direction of those effects being mixed, even within species (Fig 4; Table 4).

Most plants, across treatments and species, exhibited moderate levels of leaf chlorosis (i.e., 26–50%), with relatively few observed cases of milder (i.e., 0–25%) or more pronounced (51–100%) chlorosis (Fig 5). *Dianthus* exhibited somewhat higher chlorosis levels than other species, while *T*. *violacea* exhibited notably lower levels of chlorosis. As with wilting, RDX soil concentration was, by far, the most common factor in significant differences in chlorosis levels among treatments. In general, RDX soil contamination increased levels of chlorosis, with little difference in the effects of the two different RDX concentrations. Water-resourcing did not appear to influence levels of chlorosis. Of particular interest, significant interaction effects were observed in *R*. *caroliniensis* and *T*. *violacea*. In both cases, the strength or direction of the interaction effect on chlorosis was mixed.

In addition to experimentally testing for effects of different dual-factor treatments on common plant health indices like wilting and chlorosis, a distinctive feature of our study included an attempt to gauge the impacts of these treatments on chlorophyll levels in leaf tissues. This was done through measurements of a chlorophyll context index (*CCI*). Though similar metrics have been used in a few other RDX phytotoxicity studies [2, 43], to the best of our knowledge, *CCI* has not been previously used to gauge the plant health impacts of RDX or other munitions.

*CCI* varied widely between plants, but there were very few significant differences among factors or treatment groups (Fig 6). RDX soil concentration was a factor associated with significant differences in $\bar{CCI}$ for one species (*Dianthus* sp.), as was water-resourcing (*P*. *auriculata*). Interestingly, the lower water-resourcing level was associated with higher $\bar{CCI}$ in *P*. *auriculata* (Fig 6). There were no other significant associations between this water-resourcing level and relatively better health in this species, though a larger sample size may have supported an association with a reduced level of chlorosis (Figs 4 and 5). It would be tempting to assume that the 0.5X water-resourcing level was optimal for *P*. *auriculate*, however the 1X | 0 ppm treatment groups exhibited the lowest (or among the lowest) levels of wilting and chlorosis within this species. There were no significant factor interaction effects on $\bar{CCI}$ for any of the tested species.

#### Perspectives from greenhouse trial

The dynamics of RDX uptake, root bioaccumulation, and plant health within RDX contaminated sites are clearly complex. Predictions regarding soil contaminant uptake and fate in plants, as well as predicted plant health responses to RDX soil contamination, will, not unexpectedly, be insufficient when based on a single factor. The trend for greater proportional reduction in soil RDX when RDX concentrations are higher (Fig 2), with some interaction effect associated with water availability, has implications for phytoremediation practices, and, perhaps, particularly for in situ phytoremediation (i.e., occurring in place on contaminated soils) in regions that may experience reduced or more variable precipitation in the future. *PRC*s in the nonnative (relative to the southeastern U.S.) plant *P*. *auriculata* were relatively high and notably consistent, regardless of starting soil RDX concentrations or water-resourcing (Fig 2). Ad hoc analysis showed that the among treatment mean *PRC* in this species (${\bar{PRC}}_{PLAU}=61.2\%$) was 12% greater than the mean of corresponding values among all other plants ($\bar{PRC}=54.3\%,$ SD = 8.2%), and that the among-treatment variance in this species (*σ*^2^_*PRC∙PLAU*_ = 0.2%) was only 2.5% of the mean of corresponding values seen in other plants ($\bar{\sigma_{PRC}^{2}}$ = 7.7%, SD = 4.5%). A southeastern U.S. native plant, *S*. *coccinea*, exhibited markedly consistent levels of mean *CCI* among treatments ($\sigma_{\bar{CCI∙}SACO}^{2}$ = 0.67) regardless of starting RDX concentrations in the soil or water-resourcing. Variance within *S*. *coccinea* was only 6.5% of the mean of corresponding values seen in other plants ($\bar{{\sigma^{2}}_{\bar{CCI}}}$ = 10.02, SD = 13.24). Both of these species also exhibited relatively low among-treatment levels of mean wilting ($\bar{W_{PLAU}}=0.74,{SD}_{PLAU}=0.27;\;\bar{W_{SACO}}=0.39,{SD}_{SACO}=0.31$) and chlorosis ($\bar{C_{PLAU}}=0.92,{SD}_{PLAU}=0.50;\;\bar{C_{SACO}}=1.15,{SD}_{SACO}=0.22$). These values were and 66% and 35%, and 67% and 84% of the mean of corresponding values seen in other plants ($\bar{W}=1.13,SD=0.54;\;\bar{C}=1.37,SD=0.54$), respectively. These observations point to these plants being promising candidates for further study as robust, reliable resources for in situ phytoremediation in their native region. Though not a native species, *P*. *auriculata* has become naturalized in several southeastern U.S. states (indicating a potential for acclimation to some sites within region), and has not been observed to be aggressively invasive [44]. The high variability in health effects observed across species and between treatments also reinforces the benefits to phytoremediation efforts of local pilot studies. Such studies would help overcome some of the inherent unpredictability of how plants will respond to different combinations of environmental factors.

### Outdoor plot trial: RDX concentrations and plant survival

**Fig 7:**
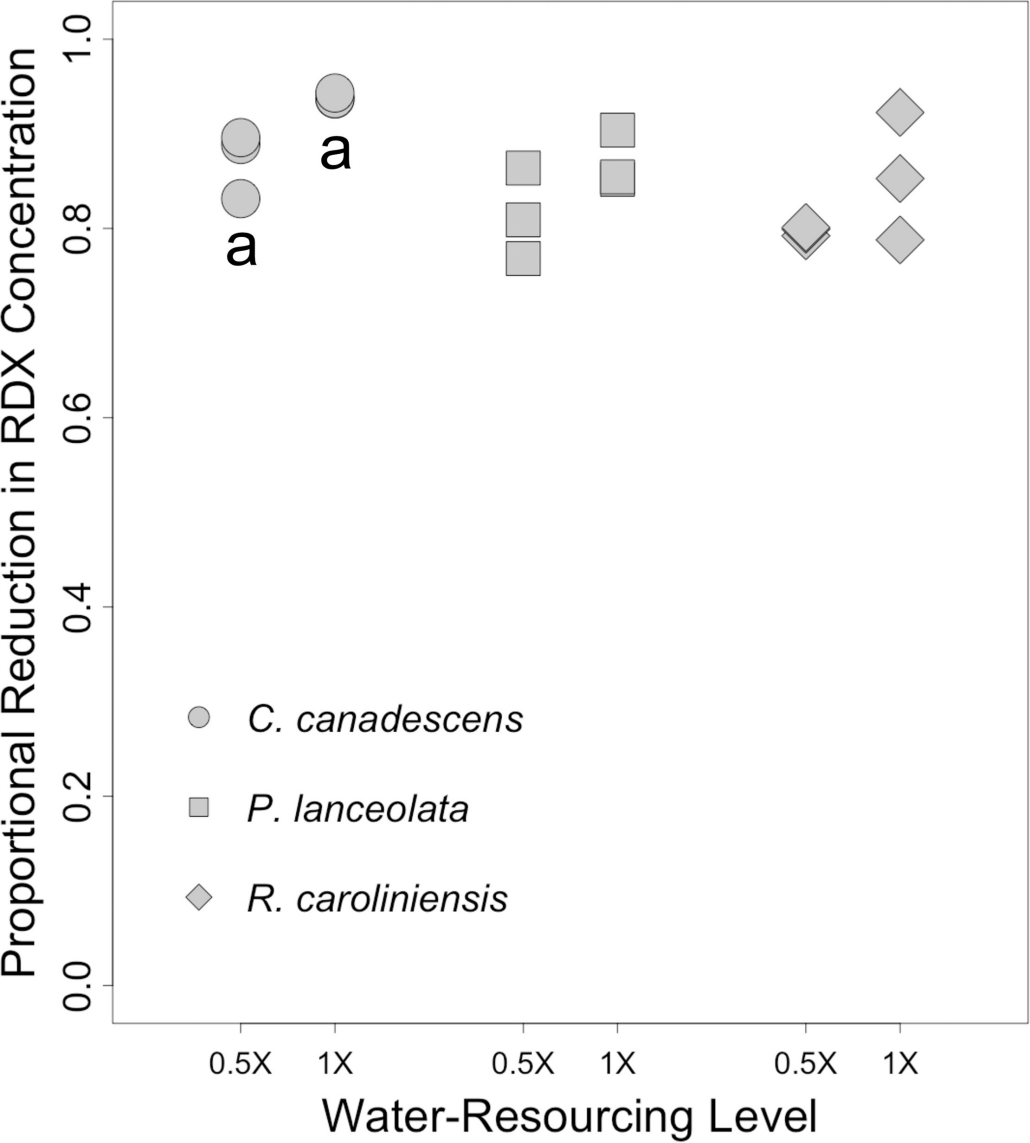
Proportional reductions in soil RDX concentrations in outdoor trials. Proportional reductions in concentration (*PRC*s) in soils from pots containing three different plant species maintained outdoors with either a 1X or 0.5X water-resourcing treatment for at least 41 days. Initial concentration of RDX in potting soils was 100 ppm. For each species, soils from n = 3 plants were sampled per treatment group. a = significant pairwise difference between treatment groups (*p* ≤ 0.10).

As evidenced by *PRC* levels, at the end of the outdoor trial, RDX soil concentrations were much lower than the starting concentration of 100 ppm (Fig 7). *PRC*s were not widely divergent among species (Fig 7). Though there appeared to be a trend towards higher *PRC*s associated with 1X water-resourcing, this pattern was only statistically significant in the case of *C*. *canescens* (*p* = 0.10).

**Fig 8:**
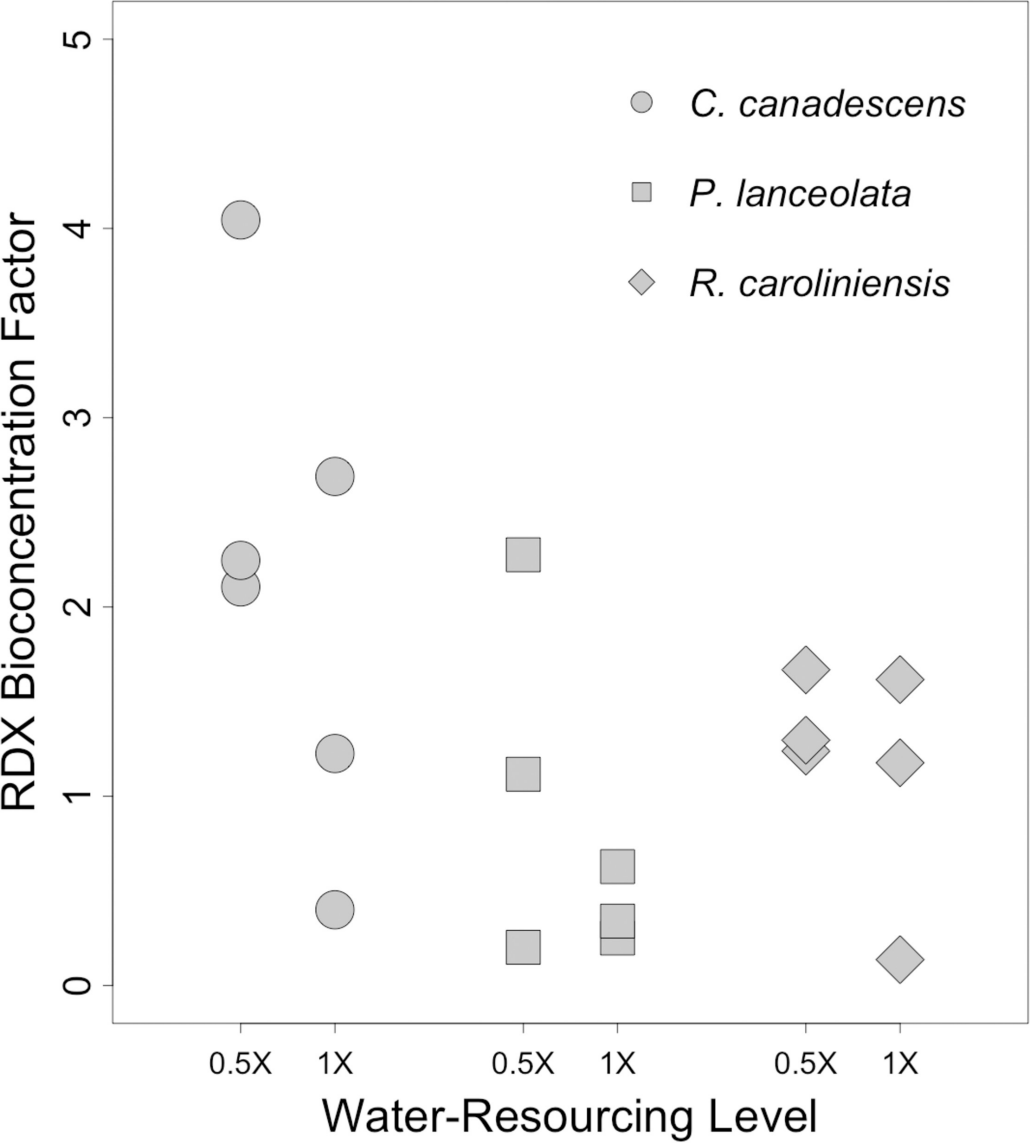
RDX bioconcentration factors in outdoor trials. Bioconcentration factors (*BCF*s) for RDX in leaf and shoot tissues of three different plant species maintained outdoors with either a 1X or 0.5X water-resourcing treatment for at least 41 days. Initial concentration of RDX in potting soils was 100 ppm. For each species, above-ground tissues from n = 3 plants were sampled per treatment group.

By the end of the trial, plants from all three species had bioaccumulated RDX (Fig 8). In most cases, *BCF*s were greater than the starting concentration of 100 ppm (*BCF* > 1). Though there appeared to be a trend of higher *BCF*s within the 0.5X water-resourcing treatment, there were no statistically significant differences among treatment groups.

**Fig 9:**
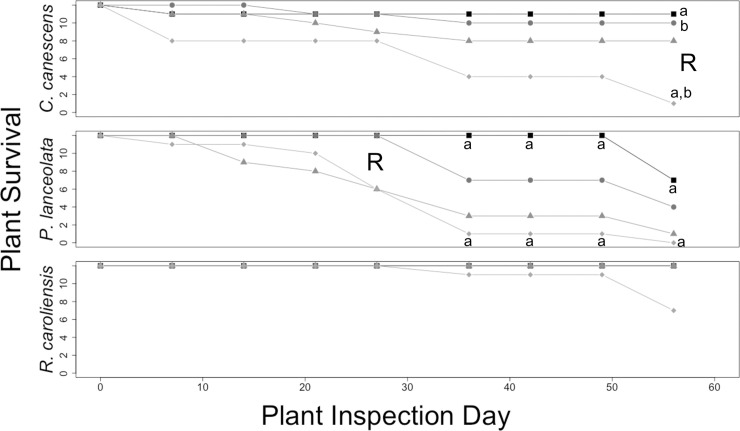
Plant survival in outdoor plot trial. Numbers of surviving *C*. *canescens*, *P*. *lanceolata*, *and R*. *caroliniensis* under four treatments over 56 days. Treatments included reduced water-resourcing (1X vs. 0.5X) and soil contamination with RDX (0 vs 100 ppm). R = day by which significant differences in survival had emerged (adjusted α = 0.05); a–b = day by which significant pairwise difference between treatment groups (designated by same letter) emerged (adjusted α = 0.017).

All three plant species exhibited different survival patterns, with RDX being the primary factor associated with plant mortality (Fig 9). In *C*. *canescens*, plant survival significantly diverged between treatments with and without RDX by Day 56 (*p* = 0.029), and *P*. *lanceolata* by Day 26 (and persisting through Day 56 (*p* = 0.002–0.046)). There was, to an extent, a combined effect between RDX concentrations and water-resourcing, as the 0.5X | 100 ppm treatment group consistently exhibited the lowest survival rate. This treatment group exhibited significantly different survival from several other treatment groups at Day 56 in *C*. *canescens* (*p* = 0.004–0.020), and from the 1X | 0 ppm treatment group in *P*. *lanceolata* starting on Day 26 (and persisting through Day 56 (*p* = 0.002–0.046)). For *R*. *caroliniensis*, only the 0.5X | 100 ppm treatment group exhibited any plant mortality, beginning between Day 27 and Day 36. There were no statistically significant differences in plant survival among treatment groups in this species.

#### Perspectives from outdoor plot trial

RDX uptake and bioaccumulation of plants under approximately natural conditions appeared to be influenced by water availability, though without stronger statistical patterns and additional, more comprehensive data (e.g., root *BCF*, changes in plant biomass), a greater understanding of the mechanisms involved is not possible. As was the case with plant health in the greenhouse trials, initial RDX concentration was the more influential factor in plant survival on outdoor plots than water-resourcing, as evidenced by the statistically significant association of plant survival with the former, but not with the latter (Fig 9). Also, as observed with plant health, RDX soil contamination and water availability appeared to have an interaction effect on plant survival in some cases (*C*. *canescens*, and, perhaps, *R*. *caroliniensis*), but not all (*P*. *lanceolata*). This highlights the fact that plant survival in RDX contaminated soil is likely a complex and difficult-to-predict phenomenon, influenced by additional factors or stressors. The southeastern U.S. native plant *R*. *caroliniensis* exhibited substantially lower mortality ($\bar{S_{RUCA}}$ = 90%, SD = 21%; Fig 9) than the other two test species ($\bar{S_{COCA}}$ = 63%, SD = 38%; $\bar{S_{PELA}}$ = 25%, SD = 26%), despite similar RDX uptake and bioconcentration levels (Figs 7 and 8). This observation indicates that this species may also be a promising candidate for further study as an in situ phytoremediation resource in its native region.

## Conclusions

In this study we demonstrated RDX uptake and bioconcentration in nine previously untested species of plants. We further experimentally demonstrated that an additional factor, water availability, could significantly change plant-RDX interactions, as demonstrated by statistically significant interaction effects between soil concentrations of RDX and water-resourcing levels. The impacts of soil RDX, water-resourcing, and interaction effects of these two factors on plant RDX uptake, bioconcentration, health, and survival were typically complex and not easily generalizable. These observations have implications for understanding how plant species (and hence, plant communities) might respond to RDX soil contamination under different climatic scenarios, and for selecting specific plant species for in situ phytoremediation of RDX.

## Supporting information

S1 File RDX concentrations and translocation factors for leaf and flower tissues in the greenhouse trial. A brief description of methods and results for RDX concentrations in several plant speciess maintained under different treatments in the greenhouse trial.
(DOCX)

S1 Table **A and B.** Leaf and flower RDX concentration data from greenhouse trial. Tables of RDX concentrations for soil (Table A, “soil_rdx”) and leaves (Table B, “leaf_rdx”) for several plant species (“plant_species”) maintained under different treatments (“treatment”) in the greenhouse trial. Treatment groups were based on different initial soil concentrations of RDX (“rdx”) and water-resourcing (“water”).
(DOCX)

S2 Table Soil and root RDX concentration data from greenhouse trial. Table of soil and root RDX concentrations (“soil_rdx,” “root_rdx,” respectively) for each individual plant (“unit”) within each treatment group (“treatment”) and within each of eight species (“plant_species”; Table 1). Treatment groups based on different initial soil concentrations of RDX (“rdx”) and water-resourcing (“water”).
(PDF)

S3 Table **A, B, and C.** Wilting, chlorosis, and chlorophyll content index data from greenhouse trial. Tables of estimated wilting levels (Table A), estimated chlorosis levels (Table B), and chlorophyll content index values (Table C) for four leaves (M1-M4) from each inidividual plant (“unit”) within each treatment group (“treatment”) and from within each of eight species (“species”; Table 1). Treatment groups were based on different initial soil concentrations of RDX (“rdx”) and water-resourcing (“water”).
(PDF)

S4 Table **A and B.** Soil and shoot RDX concentration data from outdoor plot trial. Tables of soil (Table A) and shoot (Table B) RDX concentrations (“soil_rdx,” “shoot_rdx,” respectively) for individual plants within each treatment group (“treatment”) and within each of three species (“plant_species”; Table 1). Treatment groups were based on different initial soil concentrations of RDX (“rdx”) and water-resourcing (“water”).
(PDF)
