# Supplementary material for: Multiple stressors in multiple species: Effects of different RDX soil concentrations and differential water-resourcing on RDX fate, plant health, and plant survival
Source: PLoS One. 2020 Aug 14;15(8):e0234166. doi: 10.1371/journal.pone.0234166 (PMC7428167; doi:10.1371/journal.pone.0234166)
Supplement: S1 File — A brief description of methods and results for RDX concentrations in several plant speciess maintained under different treatments in the greenhouse trial. (DOCX) [file pone.0234166.s001.docx]

**S1**

**RDX Concentrations and Translocation Factors for Leaf and Flower Tissues in the Greenhouse Trial**

Translocation factors were calculated as the ratio of RDX concentrations measured in leaf or flower tissues to RDX concentrations measured in root tissues (Table S1A, Table S1B, Table S2A). At the time of sampling for RDX concentration determination, only three species (*Dianthus, Hibiscus mocheutos, and Salvia coccinea*) still had leaves. RDX accumulated in the leaf tissues of these three plants (Table S1A) to concentrations exceeding those in the soil (TFs > 1), indicating that these were high efficiency plants for RDX translocation from roots to leaves. At the time of sampling for RDX concentration determination, only five species (*Antirrhinum majus, Pentas lanceolata, Plumbago auriculata, Ruellia caroliniensis, and Tulbaghia violacea*) still had flowers. RDX accumulated in the flower tissues of these five plants (Table S1B) at lower concentrations than those in the soil (TFs < 1), indicating low potential for RDX translocation from roots to flowers.

**Table S1A.** Final means and standard deviations of leaf RDX concentrations (ppm; Mean RDX ± SD), means and standard deviation of translocation factors (Mean TF ± SD), and sample size (N) for three plant species exposed to different treatment combinations of water-resourcing (1X or 0.5X) and initial soil RDX concentration (50 ppm or 100 ppm).

|  | Plant Species | | |
| --- | --- | --- | --- |
|  | *Dianthus* | *H. mocheutos* | *S. coccinea* |
| Treatment | Mean RDX ± SD  Mean TF ± SD  N | Mean RDX ± SD  Mean TF ± SD  N | Mean RDX ± SD  Mean TF ± SD  N |
| 1X \| 50 ppm | No sample | 774.83 ± 851.57  6.34 ± 6.93  4 | No sample |
| 0.5X \| 50 ppm | No sample | 347.70 ± 284.59  4.36 ± 2.62  5 | No sample |
| 1X \| 100 ppm | 1942.36 ± 717.82  56.07 ± 52.28  4 | No sample | 1996.61 ± 1234.27  80.59 ± 89.63  2 |
| 0.5X \| 100 ppm | No sample | 1840.98 ± 1556.19  70.77 ± 0.38  2 | No sample |

**Table S1B.** Flower RDX concentrations (ppm) and translocation factors (TF) for one plant per treatment group for each of five plant species exposed to different combinations of water-resourcing (1X or 0.5X) and initial soil RDX concentration (50 ppm or 100 ppm).

|  | Plant Species | | | | |
| --- | --- | --- | --- | --- | --- |
|  | *A. majus* | *P. lanceolata* | *P. auriculata* | *S. coccinea* | *T. violacea* |
| Treatment | RDX  TF | RDX  TF | RDX  TF | RDX  TF | RDX  TF |
| 1X \| 50 ppm | 5.55  0.06 | 3.56  0.01 | 1.65  < 0.01 | No sample | 38.22  0.13 |
| 0.5X \| 50 ppm | No sample | 15.46  0.16 | 8.24  0.03 | No sample | 16.94  0.09 |
| 1X \| 100 ppm | 3.00  0.04 | No sample | 5.19  0.05 | 11.07  0.17 | 26.87  0.17 |
| 0.5X \| 100 ppm | 36.22  0.36 | 38.80  0.21 | 10.08  0.21 | No sample | 47.89  0.39 |
