# Supplementary material for: Multiple stressors in multiple species: Effects of different RDX soil concentrations and differential water-resourcing on RDX fate, plant health, and plant survival
Source: PLoS One. 2020 Aug 14;15(8):e0234166. doi: 10.1371/journal.pone.0234166 (PMC7428167; doi:10.1371/journal.pone.0234166)
Supplement: S1 Table — A and B. Leaf and flower RDX concentration data from greenhouse trial. Tables of RDX concentrations for soil (Table A, “soil_rdx”) and leaves (Table B, “leaf_rdx”) for several plant species (“plant_species”) maintained under different treatments (“treatment”) in the greenhouse trial. Treatment groups were based on different initial soil concentrations of RDX (“rdx”) and water-resourcing (“water”). (DOCX) [file pone.0234166.s002.docx]

**S1**

**Leaf and Flower RDX Concentration Data from Greenhouse Trial**

**Table S1A.** Leaf RDX concentrations (ppm) for individual plants within each treatment group (water-resourcing level | initial soil RDX concentration) for four plant species (*Antirrhinum majus, Dianthus, Hibiscus mocheutos,* and *Salvia coccinea*).

| **Plant Species** | **Treatment Group** | **Water** | **RDX** | **Leaf RDX (ppm)** |
| --- | --- | --- | --- | --- |
| *A. majus* | 1X\|RDX-100 | 1X | 100 | 23.00 |
| *Dianthus* | 1X\|RDX-100 | 1X | 100 | 1134.46 |
| *Dianthus* | 1X\|RDX-100 | 1X | 100 | 2572.36 |
| *Dianthus* | 1X\|RDX-100 | 1X | 100 | 1541.23 |
| *Dianthus* | 1X\|RDX-100 | 1X | 100 | 2521.40 |
| *H. mocheutos* | 0.5X\|RDX-100 | 0.5X | 100 | 740.58 |
| *H. mocheutos* | 0.5X\|RDX-100 | 0.5X | 100 | 2941.37 |
| *H. mocheutos* | 0.5X\|RDX-50 | 0.5X | 50 | 172.28 |
| *H. mocheutos* | 0.5X\|RDX-50 | 0.5X | 50 | 662.48 |
| *H. mocheutos* | 0.5X\|RDX-50 | 0.5X | 50 | 46.75 |
| *H. mocheutos* | 0.5X\|RDX-50 | 0.5X | 50 | 641.20 |
| *H. mocheutos* | 0.5X\|RDX-50 | 0.5X | 50 | 215.81 |
| *H. mocheutos* | 1X\|RDX-50 | 1X | 50 | 2025.87 |
| *H. mocheutos* | 1X\|RDX-50 | 1X | 50 | 426.92 |
| *H. mocheutos* | 1X\|RDX-50 | 1X | 50 | 121.32 |
| *H. mocheutos* | 1X\|RDX-50 | 1X | 50 | 525.21 |
| *S. coccinea* | 1X\|RDX-100 | 1X | 100 | 2869.37 |
| *S. coccinea* | 1X\|RDX-100 | 1X | 100 | 1123.85 |

**Table S1B.** Flower petal RDX concentrations (ppm) for individual plants within each treatment group (water-resourcing level | initial soil RDX concentration) for four plant species (*Antirrhinum majus, Dianthus, Hibiscus mocheutos,* and *Salvia coccinea*).

| **Plant Species** | **Treatment Group** | **Water** | **RDX** | **Flower RDX (ppm)** |
| --- | --- | --- | --- | --- |
| *A. majus* | 0.5X\|RDX-100 | 1X | 50 | 36.22 |
| *A. majus* | 1X\|RDX-100 | 0.5X | 100 | 8.19 |
| *A. majus* | 1X\|RDX-50 | 1X | 100 | 5.55 |
| *P. lanceolata* | 0.5X\|RDX-100 | 1X | 50 | 38.80 |
| *P. lanceolata* | 0.5X\|RDX-50 | 0.5X | 50 | 15.46 |
| *P. lanceolata* | 1X\|RDX-50 | 1X | 100 | 3.56 |
| *P. auriculata* | 0.5X\|RDX-100 | 1X | 50 | 10.08 |
| *P. auriculata* | 0.5X\|RDX-50 | 0.5X | 50 | 8.24 |
| *P. auriculata* | 1X\|RDX-100 | 0.5X | 50 | 5.19 |
| *P. auriculata* | 1X\|RDX-50 | 1X | 100 | 1.65 |
| *S. coccinea* | 1X\|RDX-100 | 0.5X | 50 | 11.07 |
| *T. violacea* | 0.5X\|RDX-100 | 1X | 50 | 47.89 |
| *T. violacea* | 0.5X\|RDX-50 | 0.5X | 50 | 16.94 |
| *T. violacea* | 1X\|RDX-100 | 0.5X | 100 | 26.87 |
| *T. violacea* | 1X\|RDX-50 | 1X | 100 | 38.22 |
