# Supplementary material for: Multiple stressors in multiple species: Effects of different RDX soil concentrations and differential water-resourcing on RDX fate, plant health, and plant survival
Source: PLoS One. 2020 Aug 14;15(8):e0234166. doi: 10.1371/journal.pone.0234166 (PMC7428167; doi:10.1371/journal.pone.0234166)
Supplement: S2 Table — Table of soil and root RDX concentrations (“soil_rdx,” “root_rdx,” respectively) for each individual plant (“unit”) within each treatment group (“treatment”) and within each of eight species (“plant_species”; Table 1). Treatment groups based on different initial soil concentrations of RDX (“rdx”) and water-resourcing (“water”). (PDF) [file pone.0234166.s003.pdf]

S2
Soil and Root RDX Concentrations from the Greenhouse Trial

Table S2A. Soil and root RDX concentrations (ppm) for each individual plant unit within each treatment group (water-resourcing level | initial soil RDX concentration) for eight plant species (*Antirrhinum majus, Dianthus, Hibiscus mocheutos, Plumbago auriculata, Pentas lanceolata, Ruellia caroliniensis, Salvia coccinea, and Tulbaghia violacea*).

Plant Species	Treatment Group	Unit	Soil RDX (ppm)	Root RDX (ppm)
A. majus	0.5X | 100 ppm	1	27.45	101.75
A. majus	0.5X | 100 ppm	2	3.97	183.30
A. majus	0.5X | 100 ppm	3	12.24	89.72
A. majus	0.5X | 100 ppm	4	18.15	59.59
A. majus	0.5X | 100 ppm	5	23.89	95.39
A. majus	0.5X | 50 ppm	2	60.72	42.77
A. majus	0.5X | 50 ppm	3	40.79	161.17
A. majus	0.5X | 50 ppm	5	14.09	93.55
A. majus	1X | 100 ppm	2	35.01	72.84
A. majus	1X | 100 ppm	3	29.67	81.16
A. majus	1X | 100 ppm	4	19.04	0.00
A. majus	1X | 100 ppm	5	52.21	87.91
A. majus	1X | 50 ppm	2	24.58	268.33
A. majus	1X | 50 ppm	3	35.63	61.60
A. majus	1X | 50 ppm	4	45.36	97.14
Dianthus	0.5X | 100 ppm	1	28.73	63.63
Dianthus	0.5X | 100 ppm	2	5.10	47.00
Dianthus	0.5X | 100 ppm	3	12.32	95.16
Dianthus	0.5X | 100 ppm	4	12.87	65.86
Dianthus	0.5X | 100 ppm	5	3.13	81.36
Dianthus	0.5X | 50 ppm	1	4.87	30.78
Dianthus	0.5X | 50 ppm	2	20.62	89.32
Dianthus	0.5X | 50 ppm	3	16.17	53.63
Dianthus	0.5X | 50 ppm	4	12.43	20.52
Dianthus	0.5X | 50 ppm	5	13.46	45.57
Dianthus	1X | 100 ppm	1	3.26	82.97
Dianthus	1X | 100 ppm	2	42.50	23.70
Dianthus	1X | 100 ppm	3	84.57	12.07
Dianthus	1X | 100 ppm	4	41.34	16.49
Dianthus	1X | 100 ppm	5	38.99	292.38
Dianthus	1X | 50 ppm	1	11.84	23.28
Dianthus	1X | 50 ppm	2	3.81	37.65
Dianthus	1X | 50 ppm	3	54.28	167.88
Dianthus	1X | 50 ppm	4	27.19	32.82
Dianthus	1X | 50 ppm	5	29.16	51.47
H. mocheutos	0.5X | 100 ppm	1	40.77	33.61
H. mocheutos	0.5X | 100 ppm	2	18.54	60.33
H. mocheutos	0.5X | 100 ppm	3	13.42	18.71

H. mocheutos	0.5X | 100 ppm	4	31.71	41.41
H. mocheutos	0.5X | 100 ppm	5	36.12	10.50
H. mocheutos	0.5X | 50 ppm	1	5.38	45.68
H. mocheutos	0.5X | 50 ppm	2	71.04	84.15
H. mocheutos	0.5X | 50 ppm	3	52.09	59.99
H. mocheutos	0.5X | 50 ppm	4	46.03	113.88
H. mocheutos	0.5X | 50 ppm	5	4.61	57.61
H. mocheutos	1X | 100 ppm	1	23.13	124.82
H. mocheutos	1X | 100 ppm	2	48.12	97.90
H. mocheutos	1X | 100 ppm	3	60.78	186.45
H. mocheutos	1X | 100 ppm	4	40.23	88.39
H. mocheutos	1X | 100 ppm	5	39.79	94.31
H. mocheutos	1X | 50 ppm	1	45.70	84.82
H. mocheutos	1X | 50 ppm	2	24.74	123.73
H. mocheutos	1X | 50 ppm	3	24.49	85.25
H. mocheutos	1X | 50 ppm	4	28.94	203.30
H. mocheutos	1X | 50 ppm	5	37.35	154.79
P. auriculata	0.5X | 100 ppm	1	33.29	No Data
P. auriculata	0.5X | 100 ppm	2	28.58	48.67
P. auriculata	0.5X | 100 ppm	3	46.08	68.37
P. auriculata	0.5X | 50 ppm	1	7.16	264.51
P. auriculata	0.5X | 50 ppm	2	18.42	65.47
P. auriculata	0.5X | 50 ppm	3	38.49	142.23
P. auriculata	1X | 100 ppm	1	62.20	35.56
P. auriculata	1X | 100 ppm	2	9.63	16.89
P. auriculata	1X | 100 ppm	3	29.85	97.31
P. auriculata	1X | 50 ppm	1	9.14	30.18
P. auriculata	1X | 50 ppm	2	24.15	5093.00
P. auriculata	1X | 50 ppm	3	30.50	3912.09
P. lanceolata	0.5X | 100 ppm	1	5.49	47.67
P. lanceolata	0.5X | 100 ppm	2	7.69	183.52
P. lanceolata	0.5X | 100 ppm	3	28.01	54.54
P. lanceolata	0.5X | 100 ppm	4	10.77	77.72
P. lanceolata	0.5X | 100 ppm	5	6.38	114.27
P. lanceolata	0.5X | 50 ppm	1	55.70	77.26
P. lanceolata	0.5X | 50 ppm	2	60.03	97.21
P. lanceolata	0.5X | 50 ppm	3	43.86	69.84
P. lanceolata	0.5X | 50 ppm	4	6.64	68.80
P. lanceolata	0.5X | 50 ppm	5	37.46	42.71
P. lanceolata	1X | 100 ppm	1	86.77	96.50
P. lanceolata	1X | 100 ppm	2	30.19	83.72
P. lanceolata	1X | 100 ppm	3	3.88	226.37
P. lanceolata	1X | 100 ppm	5	16.03	366.45
P. lanceolata	1X | 50 ppm	1	29.22	452.32
P. lanceolata	1X | 50 ppm	2	19.11	239.07
P. lanceolata	1X | 50 ppm	3	44.48	1509.33

P. lanceolata	1X | 50 ppm	4	47.92	68.89
P. lanceolata	1X | 50 ppm	5	24.71	1377.58
R. caroliniensis	0.5X | 100 ppm	1	16.81	23.89
R. caroliniensis	0.5X | 100 ppm	2	7.13	13.82
R. caroliniensis	0.5X | 100 ppm	3	No Data	32.98
R. caroliniensis	0.5X | 50 ppm	1	49.30	16.04
R. caroliniensis	0.5X | 50 ppm	2	42.72	29.23
R. caroliniensis	0.5X | 50 ppm	3	70.28	36.30
R. caroliniensis	0.5X | 50 ppm	4	No Data	18.26
R. caroliniensis	1X | 100 ppm	1	42.11	88.53
R. caroliniensis	1X | 100 ppm	2	54.33	35.94
R. caroliniensis	1X | 100 ppm	3	47.91	59.58
R. caroliniensis	1X | 100 ppm	4	25.68	70.91
R. caroliniensis	1X | 50 ppm	1	50.54	13.29
R. caroliniensis	1X | 50 ppm	2	8.40	20.48
R. caroliniensis	1X | 50 ppm	3	12.34	15.47
R. caroliniensis	1X | 50 ppm	4	18.84	17.48
S. coccinea	0.5X | 100 ppm	1	82.30	32.35
S. coccinea	0.5X | 100 ppm	2	8.23	35.18
S. coccinea	0.5X | 100 ppm	3	28.27	49.14
S. coccinea	0.5X | 100 ppm	4	2.62	24.09
S. coccinea	0.5X | 100 ppm	5	46.32	25.03
S. coccinea	0.5X | 50 ppm	1	35.50	24.85
S. coccinea	0.5X | 50 ppm	2	59.79	29.33
S. coccinea	0.5X | 50 ppm	3	52.38	72.79
S. coccinea	0.5X | 50 ppm	4	47.64	26.79
S. coccinea	0.5X | 50 ppm	5	25.24	12.58
S. coccinea	1X | 100 ppm	1	44.02	19.93
S. coccinea	1X | 100 ppm	2	42.12	65.29
S. coccinea	1X | 100 ppm	3	71.01	37.60
S. coccinea	1X | 100 ppm	4	60.01	32.44
S. coccinea	1X | 50 ppm	1	41.87	21.84
S. coccinea	1X | 50 ppm	2	20.49	23.30
S. coccinea	1X | 50 ppm	3	19.28	29.11
S. coccinea	1X | 50 ppm	4	26.81	21.36
S. coccinea	1X | 50 ppm	5	10.26	38.00
T. violacea	0.5X | 100 ppm	1	9.55	124.12
T. violacea	0.5X | 100 ppm	2	No Data	183.36
T. violacea	0.5X | 100 ppm	3	No Data	95.31
T. violacea	0.5X | 50 ppm	1	No Data	55.97
T. violacea	0.5X | 50 ppm	2	43.36	108.03
T. violacea	0.5X | 50 ppm	3	4.66	180.77
T. violacea	1X | 100 ppm	1	26.31	11.08
T. violacea	1X | 100 ppm	2	28.75	162.81
T. violacea	1X | 100 ppm	3	39.45	111.04
T. violacea	1X | 50 ppm	1	12.27	95.34

T. violacea	1X | 50 ppm	2	6.75	301.19
T. violacea	1X | 50 ppm	3	9.85	69.85
