# Supplementary material for: Multiple stressors in multiple species: Effects of different RDX soil concentrations and differential water-resourcing on RDX fate, plant health, and plant survival
Source: PLoS One. 2020 Aug 14;15(8):e0234166. doi: 10.1371/journal.pone.0234166 (PMC7428167; doi:10.1371/journal.pone.0234166)
Supplement: S3 Table — A, B, and C. Wilting, chlorosis, and chlorophyll content index data from greenhouse trial. Tables of estimated wilting levels (Table A), estimated chlorosis levels (Table B), and chlorophyll content index values (Table C) for four leaves (M1-M4) from each inidividual plant (“unit”) within each treatment group (“treatment”) and from within each of eight species (“species”; Table 1). Treatment groups were based on different initial soil concentrations of RDX (“rdx”) and water-resourcing (“water”). (PDF) [file pone.0234166.s004.pdf]

S3
Wilting, Chlorosis, and Chlorophyll Content Index Data from the Greenhouse Trial

Table S3A. Estimated wilting levels (ranked 1-4, corresponding to 1–25%, 26–50%, 51–75%, 76–100% wilted, respectively) for four leaves (L1-L4) from each inidividual plant unit within each treatment group (water-resourcing level | initial soil RDX concentration) for eight plant species (*Antirrhinum majus, Dianthus, Hibiscus mocheutos, Plumbago auriculata, Pentas lanceolata, Ruellia caroliniensis, Salvia coccinea, and Tulbaghia violacea*).

			**Wilt Level**			
**Plant Species**	**Treatment Group**	**Unit**	**L1**	**L2**	**L3**	**L4**
A. majus	0.5X | 0 ppm	1	1	1	1	1
A. majus	0.5X | 0 ppm	2	1	1	1	1
A. majus	0.5X | 0 ppm	3	2	2	2	3
A. majus	0.5X | 0 ppm	4	2	2	1	1
A. majus	0.5X | 0 ppm	5	1	1	1	1
A. majus	0.5X | 100 ppm	1	1	1	2	1
A. majus	0.5X | 100 ppm	2	1	1	1	1
A. majus	0.5X | 100 ppm	3	1	1	1	2
A. majus	0.5X | 100 ppm	4	1	1	1	1
A. majus	0.5X | 100 ppm	5	1	1	1	1
A. majus	0.5X | 50 ppm	1	1	1	1	1
A. majus	0.5X | 50 ppm	2	1	1	2	1
A. majus	0.5X | 50 ppm	3	1	1	1	1
A. majus	0.5X | 50 ppm	4	2	2	1	1
A. majus	0.5X | 50 ppm	5	1	1	1	1
A. majus	1X | 0 ppm	1	1	1	1	1
A. majus	1X | 0 ppm	2	1	1	1	1
A. majus	1X | 0 ppm	3	1	1	1	1
A. majus	1X | 0 ppm	4	1	1	1	1
A. majus	1X | 0 ppm	5	1	1	1	1
A. majus	1X | 100 ppm	1	1	1	1	1
A. majus	1X | 100 ppm	2	2	1	1	1
A. majus	1X | 100 ppm	3	1	1	1	1
A. majus	1X | 100 ppm	4	1	1	1	1
A. majus	1X | 100 ppm	5	1	1	2	1
A. majus	1X | 50 ppm	1	1	1	1	1
A. majus	1X | 50 ppm	2	1	1	1	1
A. majus	1X | 50 ppm	3	1	1	1	1
A. majus	1X | 50 ppm	4	1	1	1	1
A. majus	1X | 50 ppm	5	1	1	1	1
Dianthus	0.5X | 0 ppm	1	4	2	1	4
Dianthus	0.5X | 0 ppm	2	4	1	1	1
Dianthus	0.5X | 0 ppm	3	1	1	3	1
Dianthus	0.5X | 0 ppm	4	1	1	1	1

Dianthus	0.5X | 0 ppm	5	3	3	1	1
Dianthus	0.5X | 100 ppm	1	1	1	1	1
Dianthus	0.5X | 100 ppm	2	2	2	1	1
Dianthus	0.5X | 100 ppm	3	1	4	1	1
Dianthus	0.5X | 100 ppm	4	1	2	2	1
Dianthus	0.5X | 100 ppm	5	1	1	1	4
Dianthus	0.5X | 50 ppm	1	4	4	1	5
Dianthus	0.5X | 50 ppm	2	2	4	4	1
Dianthus	0.5X | 50 ppm	3	4	2	4	4
Dianthus	0.5X | 50 ppm	4	1	1	1	1
Dianthus	0.5X | 50 ppm	5	2	1	3	4
Dianthus	1X | 0 ppm	1	1	3	1	2
Dianthus	1X | 0 ppm	2	1	1	1	1
Dianthus	1X | 0 ppm	3	4	1	1	4
Dianthus	1X | 0 ppm	4	1	1	1	2
Dianthus	1X | 0 ppm	5	3	1	2	1
Dianthus	1X | 100 ppm	1	1	1	1	1
Dianthus	1X | 100 ppm	2	1	1	3	1
Dianthus	1X | 100 ppm	3	1	1	1	4
Dianthus	1X | 100 ppm	4	1	3	1	1
Dianthus	1X | 100 ppm	5	1	1	1	1
Dianthus	1X | 50 ppm	1	4	1	1	5
Dianthus	1X | 50 ppm	2	4	4	3	3
Dianthus	1X | 50 ppm	3	4	2	4	1
Dianthus	1X | 50 ppm	4	4	3	0	4
Dianthus	1X | 50 ppm	5	1	3	1	1
H. mocheutos	0.5X | 0 ppm	1	1	1	0	1
H. mocheutos	0.5X | 0 ppm	2	1	1	1	1
H. mocheutos	0.5X | 0 ppm	3	0	1	0	0
H. mocheutos	0.5X | 0 ppm	4	1	1	0	1
H. mocheutos	0.5X | 0 ppm	5	1	0	1	1
H. mocheutos	0.5X | 100 ppm	1	1	1	1	1
H. mocheutos	0.5X | 100 ppm	2	1	1	1	1
H. mocheutos	0.5X | 100 ppm	3	1	0	1	1
H. mocheutos	0.5X | 100 ppm	4	1	1	1	1
H. mocheutos	0.5X | 100 ppm	5	1	1	1	1
H. mocheutos	0.5X | 50 ppm	1	1	1	0	0
H. mocheutos	0.5X | 50 ppm	2	1	1	1	1
H. mocheutos	0.5X | 50 ppm	3	1	1	0	0
H. mocheutos	0.5X | 50 ppm	4	1	1	1	0
H. mocheutos	0.5X | 50 ppm	5	1	0	1	0
H. mocheutos	1X | 0 ppm	1	0	0	0	1
H. mocheutos	1X | 0 ppm	2	0	0	0	0
H. mocheutos	1X | 0 ppm	3	1	0	0	0
H. mocheutos	1X | 0 ppm	4	1	0	1	0
H. mocheutos	1X | 100 ppm	1	1	1	1	2

H. mocheutos	1X | 100 ppm	2	2	2	2	2
H. mocheutos	1X | 100 ppm	3	1	1	1	1
H. mocheutos	1X | 100 ppm	4	1	2	0	2
H. mocheutos	1X | 100 ppm	5	1	1	1	1
H. mocheutos	1X | 50 ppm	1	1	0	1	1
H. mocheutos	1X | 50 ppm	2	1	1	1	2
H. mocheutos	1X | 50 ppm	3	1	1	1	1
H. mocheutos	1X | 50 ppm	4	1	1	1	1
H. mocheutos	1X | 50 ppm	5	1	0	1	1
P. auriculata	0.5X | 0 ppm	1	0	1	1	1
P. auriculata	0.5X | 0 ppm	2	1	1	1	0
P. auriculata	0.5X | 100 ppm	1	1	0	1	1
P. auriculata	0.5X | 100 ppm	2	0	1	1	1
P. auriculata	0.5X | 100 ppm	3	0	0	1	0
P. auriculata	0.5X | 50 ppm	1	1	1	1	1
P. auriculata	0.5X | 50 ppm	2	1	1	1	1
P. auriculata	0.5X | 50 ppm	3	1	1	1	1
P. auriculata	1X | 0 ppm	1	0	1	0	0
P. auriculata	1X | 0 ppm	2	0	1	1	1
P. auriculata	1X | 100 ppm	1	1	0	1	0
P. auriculata	1X | 100 ppm	2	0	1	0	0
P. auriculata	1X | 100 ppm	3	0	1	1	1
P. auriculata	1X | 50 ppm	1	1	1	1	1
P. auriculata	1X | 50 ppm	2	1	2	1	1
P. auriculata	1X | 50 ppm	3		1	1	0
P. lanceolata	0.5X | 0 ppm	1	2	1	1	2
P. lanceolata	0.5X | 0 ppm	2	1	1	1	1
P. lanceolata	0.5X | 0 ppm	3	1	1	1	1
P. lanceolata	0.5X | 0 ppm	4	1	1	2	1
P. lanceolata	0.5X | 0 ppm	5	1	1	1	1
P. lanceolata	0.5X | 100 ppm	1	1	1	1	1
P. lanceolata	0.5X | 100 ppm	2	1	0	0	0
P. lanceolata	0.5X | 100 ppm	3	1	0	0	0
P. lanceolata	0.5X | 100 ppm	4	0	0	1	0
P. lanceolata	0.5X | 100 ppm	5	1	1	0	1
P. lanceolata	0.5X | 50 ppm	1	1	1	1	1
P. lanceolata	0.5X | 50 ppm	2	3	3	3	3
P. lanceolata	0.5X | 50 ppm	3	3	1	2	2
P. lanceolata	0.5X | 50 ppm	4	1	4	2	2
P. lanceolata	0.5X | 50 ppm	5	2	2	3	3
P. lanceolata	1X | 0 ppm	1	1	1	1	1
P. lanceolata	1X | 0 ppm	2	1	1	1	1
P. lanceolata	1X | 0 ppm	3	1	1	1	1
P. lanceolata	1X | 0 ppm	4	1	1	1	1
P. lanceolata	1X | 0 ppm	5	1	1	2	3
P. lanceolata	1X | 100 ppm	1	3	3	3	3

P. lanceolata	1X | 100 ppm	2	4	2	2	2
P. lanceolata	1X | 100 ppm	3	2	3	2	1
P. lanceolata	1X | 100 ppm	4	2	2	1	3
P. lanceolata	1X | 100 ppm	5	2	1	1	2
P. lanceolata	1X | 50 ppm	1	2	2	1	1
P. lanceolata	1X | 50 ppm	2	1	2	1	1
P. lanceolata	1X | 50 ppm	3	2	1	3	1
P. lanceolata	1X | 50 ppm	4	1	2	2	1
P. lanceolata	1X | 50 ppm	5	1	1	3	1
R. caroliniensis	0.5X | 0 ppm	1	1	1	1	1
R. caroliniensis	0.5X | 0 ppm	2	1	1	1	1
R. caroliniensis	0.5X | 0 ppm	3	1	1	1	1
R. caroliniensis	0.5X | 0 ppm	4	1	1	1	1
R. caroliniensis	0.5X | 100 ppm	1	1	0	1	1
R. caroliniensis	0.5X | 100 ppm	2	1	0	0	0
R. caroliniensis	0.5X | 100 ppm	3	1	1	0	0
R. caroliniensis	0.5X | 100 ppm	4	1	1	1	1
R. caroliniensis	0.5X | 50 ppm	1	1	2	1	1
R. caroliniensis	0.5X | 50 ppm	2	1	1	1	1
R. caroliniensis	0.5X | 50 ppm	3	1	1	1	1
R. caroliniensis	0.5X | 50 ppm	4	1	1	1	1
R. caroliniensis	1X | 0 ppm	1	1	1	1	1
R. caroliniensis	1X | 0 ppm	2	1	1	1	1
R. caroliniensis	1X | 0 ppm	3	1	1	1	1
R. caroliniensis	1X | 100 ppm	1	1	0	0	0
R. caroliniensis	1X | 100 ppm	2	0	0	1	0
R. caroliniensis	1X | 100 ppm	3	1	0	0	0
R. caroliniensis	1X | 100 ppm	4	0	0	0	0
R. caroliniensis	1X | 50 ppm	1	1	1	1	1
R. caroliniensis	1X | 50 ppm	2	1	1	1	1
R. caroliniensis	1X | 50 ppm	3	3	1	1	1
R. caroliniensis	1X | 50 ppm	4	1	1	1	1
S. coccinea	0.5X | 0 ppm	1	0	1	1	0
S. coccinea	0.5X | 0 ppm	2	0	0	0	0
S. coccinea	0.5X | 0 ppm	3	0	1	0	0
S. coccinea	0.5X | 0 ppm	4	0	0	0	0
S. coccinea	0.5X | 0 ppm	5	0	0	0	0
S. coccinea	0.5X | 100 ppm	1	1	0	0	0
S. coccinea	0.5X | 100 ppm	2	0	0	0	0
S. coccinea	0.5X | 100 ppm	3	0	0	0	0
S. coccinea	0.5X | 100 ppm	4	0	0	0	0
S. coccinea	0.5X | 100 ppm	5	0	0	0	0
S. coccinea	0.5X | 50 ppm	1	1	1	1	1
S. coccinea	0.5X | 50 ppm	2	1	0	1	0
S. coccinea	0.5X | 50 ppm	3	1	1	1	1
S. coccinea	0.5X | 50 ppm	4	1	1	0	1

S. coccinea	0.5X | 50 ppm	5	1	1	1	0
S. coccinea	1X | 0 ppm	1	1	0	0	1
S. coccinea	1X | 0 ppm	2	0	0	0	1
S. coccinea	1X | 0 ppm	3	0	0	0	0
S. coccinea	1X | 0 ppm	4	0	1	0	0
S. coccinea	1X | 0 ppm	5	0	0	0	0
S. coccinea	1X | 100 ppm	1	0	1	1	0
S. coccinea	1X | 100 ppm	2	1	1	0	0
S. coccinea	1X | 100 ppm	3	0	0	0	0
S. coccinea	1X | 100 ppm	4	0	1	1	1
S. coccinea	1X | 50 ppm	1	1	1	0	1
S. coccinea	1X | 50 ppm	2	1	1	1	1
S. coccinea	1X | 50 ppm	3	1	0	0	1
S. coccinea	1X | 50 ppm	4	0	1	1	0
S. coccinea	1X | 50 ppm	5	0	1	1	1
T. violacea	0.5X | 0 ppm	1	1	1	1	1
T. violacea	0.5X | 0 ppm	2	0	0	1	1
T. violacea	0.5X | 0 ppm	3	1	0	0	1
T. violacea	0.5X | 0 ppm	4	0	0	0	1
T. violacea	0.5X | 100 ppm	1	0	0	1	0
T. violacea	0.5X | 100 ppm	2	0	0	0	1
T. violacea	0.5X | 100 ppm	3	1	1	1	1
T. violacea	0.5X | 50 ppm	1	1	1	1	1
T. violacea	0.5X | 50 ppm	2	2	1	1	1
T. violacea	0.5X | 50 ppm	3	1	1	1	1
T. violacea	1X | 0 ppm	1	0	0	1	1
T. violacea	1X | 0 ppm	2	1	0	1	0
T. violacea	1X | 0 ppm	3	0	0	0	0
T. violacea	1X | 0 ppm	4	0	0	0	1
T. violacea	1X | 100 ppm	1	1	1	0	1
T. violacea	1X | 100 ppm	2	0	1	0	1
T. violacea	1X | 100 ppm	3	0	0	0	0
T. violacea	1X | 50 ppm	1	1	0	1	0
T. violacea	1X | 50 ppm	2	0	1	1	0

Table S3B. Estimated chlorosis levels (ranked 1-4, corresponding to 1–25%, 26–50%, 51–75%, 76–100% chlorosis, respectively) for four leaves (L1-L4) from each inidividual plant unit within each treatment group (water-resourcing level | initial soil RDX concentration) for eight plant species (*Antirrhinum majus, Dianthus, Hibiscus mocheutos, Plumbago auriculata, Pentas lanceolata, Ruellia caroliniensis, Salvia coccinea, and Tulbaghia violacea*).

			Chlorosis Level			
**Plant Species**	**Treatment Group**	**Unit**	**L1**	**L2**	**L3**	**L4**
A. majus	0.5X | 0 ppm	1	1	1	1	1
A. majus	0.5X | 0 ppm	2	1	1	1	1
A. majus	0.5X | 0 ppm	3	1	1	1	1
A. majus	0.5X | 0 ppm	4	2	1	1	1
A. majus	0.5X | 0 ppm	5	1	1	1	1
A. majus	0.5X | 100 ppm	1	1	1	1	1
A. majus	0.5X | 100 ppm	2	1	1	1	1
A. majus	0.5X | 100 ppm	3	1	1	1	1
A. majus	0.5X | 100 ppm	4	1	1	1	1
A. majus	0.5X | 100 ppm	5	1	1	1	1
A. majus	0.5X | 50 ppm	1	1	1	1	1
A. majus	0.5X | 50 ppm	2	2	2	2	1
A. majus	0.5X | 50 ppm	3	2	1	1	1
A. majus	0.5X | 50 ppm	4	1	1	1	1
A. majus	0.5X | 50 ppm	5	1	1	1	1
A. majus	1X | 0 ppm	1	1	1	1	1
A. majus	1X | 0 ppm	2	1	1	1	1
A. majus	1X | 0 ppm	3	1	1	1	1
A. majus	1X | 0 ppm	4	1	1	1	1
A. majus	1X | 0 ppm	5	1	1	1	1
A. majus	1X | 100 ppm	1	2	1	1	1
A. majus	1X | 100 ppm	2	1	1	1	1
A. majus	1X | 100 ppm	3	1	1	1	1
A. majus	1X | 100 ppm	4	1	1	1	1
A. majus	1X | 100 ppm	5	1	1	1	1
A. majus	1X | 50 ppm	1	1	1	2	2
A. majus	1X | 50 ppm	2	2	1	1	1
A. majus	1X | 50 ppm	3	1	1	1	1
A. majus	1X | 50 ppm	4	1	1	1	1
A. majus	1X | 50 ppm	5	1	1	1	1
Dianthus	0.5X | 0 ppm	1	4	2	1	4
Dianthus	0.5X | 0 ppm	2	4	1	1	1
Dianthus	0.5X | 0 ppm	3	1	1	3	1
Dianthus	0.5X | 0 ppm	4	1	1	1	1
Dianthus	0.5X | 0 ppm	5	3	3	1	1
Dianthus	0.5X | 100 ppm	1	1	1	1	1
Dianthus	0.5X | 100 ppm	2	2	2	1	1

Dianthus	0.5X | 100 ppm	3	1	4	1	1
Dianthus	0.5X | 100 ppm	4	1	2	2	1
Dianthus	0.5X | 100 ppm	5	1	1	1	2
Dianthus	0.5X | 50 ppm	1	4	4	1	5
Dianthus	0.5X | 50 ppm	2	2	4	4	1
Dianthus	0.5X | 50 ppm	3	4	2	4	4
Dianthus	0.5X | 50 ppm	4	1	1	1	1
Dianthus	0.5X | 50 ppm	5	2	1	3	5
Dianthus	1X | 0 ppm	1	1	3	1	2
Dianthus	1X | 0 ppm	2	1	1	1	1
Dianthus	1X | 0 ppm	3	4	1	1	4
Dianthus	1X | 0 ppm	4	1	1	1	2
Dianthus	1X | 0 ppm	5	3	1	3	1
Dianthus	1X | 100 ppm	1	1	1	1	1
Dianthus	1X | 100 ppm	2	1	1	3	1
Dianthus	1X | 100 ppm	3	1	1	1	4
Dianthus	1X | 100 ppm	4	1	3	1	1
Dianthus	1X | 100 ppm	5	1	1	1	1
Dianthus	1X | 50 ppm	1	5	1	1	5
Dianthus	1X | 50 ppm	2	4	4	3	3
Dianthus	1X | 50 ppm	3	4	2	4	1
Dianthus	1X | 50 ppm	4	4	3	0	4
Dianthus	1X | 50 ppm	5	1	3	1	1
H. mocheutos	0.5X | 0 ppm	1	3	2	1	2
H. mocheutos	0.5X | 0 ppm	2	2	1	2	1
H. mocheutos	0.5X | 0 ppm	3	1	2	1	1
H. mocheutos	0.5X | 0 ppm	4	1	2	1	3
H. mocheutos	0.5X | 0 ppm	5	2	1	2	2
H. mocheutos	0.5X | 100 ppm	1	2	3	2	1
H. mocheutos	0.5X | 100 ppm	2	2	1	1	1
H. mocheutos	0.5X | 100 ppm	3	2	1	1	2
H. mocheutos	0.5X | 100 ppm	4	2	2	2	1
H. mocheutos	0.5X | 100 ppm	5	2	2	2	2
H. mocheutos	0.5X | 50 ppm	1	3	3	1	2
H. mocheutos	0.5X | 50 ppm	2	2	2	2	1
H. mocheutos	0.5X | 50 ppm	3	4	1	1	1
H. mocheutos	0.5X | 50 ppm	4	2	2	2	2
H. mocheutos	0.5X | 50 ppm	5	3	2	3	2
H. mocheutos	1X | 0 ppm	1	1	1	1	1
H. mocheutos	1X | 0 ppm	2	2	2	2	2
H. mocheutos	1X | 0 ppm	3	2	1	1	2
H. mocheutos	1X | 0 ppm	4	3	3	2	3
H. mocheutos	1X | 100 ppm	1	1	2	1	2
H. mocheutos	1X | 100 ppm	2	2	1	1	2
H. mocheutos	1X | 100 ppm	3	1	2	2	2
H. mocheutos	1X | 100 ppm	4	1	2	1	2

H. mocheutos	1X | 100 ppm	5	2	2	1	1
H. mocheutos	1X | 50 ppm	1	1	2	2	3
H. mocheutos	1X | 50 ppm	2	3	2	1	2
H. mocheutos	1X | 50 ppm	3	1	2	3	1
H. mocheutos	1X | 50 ppm	4	2	2	3	3
H. mocheutos	1X | 50 ppm	5	1	2	3	2
P. auriculata	0.5X | 0 ppm	1	0	1	1	0
P. auriculata	0.5X | 0 ppm	2	0	1	1	0
P. auriculata	0.5X | 100 ppm	1	0	0	3	0
P. auriculata	0.5X | 100 ppm	2	1	1	1	2
P. auriculata	0.5X | 100 ppm	3	2	2	2	1
P. auriculata	0.5X | 50 ppm	1	1	1	1	1
P. auriculata	0.5X | 50 ppm	2	1	1	1	1
P. auriculata	0.5X | 50 ppm	3	1	1	1	1
P. auriculata	1X | 0 ppm	1	0	0	0	0
P. auriculata	1X | 0 ppm	2	0	0	1	0
P. auriculata	1X | 100 ppm	1	1	1	1	2
P. auriculata	1X | 100 ppm	2	2	1	0	2
P. auriculata	1X | 100 ppm	3	1	2	2	1
P. auriculata	1X | 50 ppm	1	1	1	1	1
P. auriculata	1X | 50 ppm	2	1	2	1	1
P. auriculata	1X | 50 ppm	3	4	1	1	0
P. lanceolata	0.5X | 0 ppm	1	2	1	1	2
P. lanceolata	0.5X | 0 ppm	2	1	1	1	1
P. lanceolata	0.5X | 0 ppm	3	1	1	1	1
P. lanceolata	0.5X | 0 ppm	4	1	1	2	1
P. lanceolata	0.5X | 0 ppm	5	1	1	1	1
P. lanceolata	0.5X | 100 ppm	1	2	2	1	2
P. lanceolata	0.5X | 100 ppm	2	1	1	1	1
P. lanceolata	0.5X | 100 ppm	3	1	0	1	1
P. lanceolata	0.5X | 100 ppm	4	0	1	1	1
P. lanceolata	0.5X | 100 ppm	5	1	3	0	2
P. lanceolata	0.5X | 50 ppm	1	1	1	1	1
P. lanceolata	0.5X | 50 ppm	2	3	1	3	1
P. lanceolata	0.5X | 50 ppm	3	1	1	1	1
P. lanceolata	0.5X | 50 ppm	4	1	4	1	1
P. lanceolata	0.5X | 50 ppm	5	1	1	2	1
P. lanceolata	1X | 0 ppm	1	1	1	1	1
P. lanceolata	1X | 0 ppm	2	1	1	1	1
P. lanceolata	1X | 0 ppm	3	1	1	1	1
P. lanceolata	1X | 0 ppm	4	1	1	1	1
P. lanceolata	1X | 0 ppm	5	1	1	2	3
P. lanceolata	1X | 100 ppm	1	2	2	2	3
P. lanceolata	1X | 100 ppm	2	4	2	1	1
P. lanceolata	1X | 100 ppm	3	1	1	1	1
P. lanceolata	1X | 100 ppm	4	2	2	1	1

P. lanceolata	1X | 100 ppm	5	1	1	1	2
P. lanceolata	1X | 50 ppm	1	2	2	1	1
P. lanceolata	1X | 50 ppm	2	1	1	1	1
P. lanceolata	1X | 50 ppm	3	2	1	2	1
P. lanceolata	1X | 50 ppm	4	1	1	1	1
P. lanceolata	1X | 50 ppm	5	1	1	2	1
R. caroliniensis	0.5X | 0 ppm	1	1	1	1	1
R. caroliniensis	0.5X | 0 ppm	2	1	1	1	1
R. caroliniensis	0.5X | 0 ppm	3	1	2	1	1
R. caroliniensis	0.5X | 0 ppm	4	1	1	1	1
R. caroliniensis	0.5X | 100 ppm	1	3	2	3	2
R. caroliniensis	0.5X | 100 ppm	2	3	2	2	2
R. caroliniensis	0.5X | 100 ppm	3	3	3	3	2
R. caroliniensis	0.5X | 100 ppm	4	1	1	3	1
R. caroliniensis	0.5X | 50 ppm	1	2	1	2	2
R. caroliniensis	0.5X | 50 ppm	2	2	1	1	2
R. caroliniensis	0.5X | 50 ppm	3	2	1	1	2
R. caroliniensis	0.5X | 50 ppm	4	1	1	1	1
R. caroliniensis	1X | 0 ppm	1	1	1	1	1
R. caroliniensis	1X | 0 ppm	2	2	1	1	1
R. caroliniensis	1X | 0 ppm	3	1	2	1	2
R. caroliniensis	1X | 100 ppm	1	1	1	1	1
R. caroliniensis	1X | 100 ppm	2	1	2	1	2
R. caroliniensis	1X | 100 ppm	3	3	3	1	2
R. caroliniensis	1X | 100 ppm	4	2	1	1	2
R. caroliniensis	1X | 50 ppm	1	2	1	1	1
R. caroliniensis	1X | 50 ppm	2	2	2	1	2
R. caroliniensis	1X | 50 ppm	3	2	2	1	2
R. caroliniensis	1X | 50 ppm	4	1	1	1	1
S. coccinea	0.5X | 0 ppm	1	1	1	1	1
S. coccinea	0.5X | 0 ppm	2	1	1	1	1
S. coccinea	0.5X | 0 ppm	3	1	1	1	1
S. coccinea	0.5X | 0 ppm	4	1	1	1	1
S. coccinea	0.5X | 0 ppm	5	1	1	1	1
S. coccinea	0.5X | 100 ppm	1	2	2	1	1
S. coccinea	0.5X | 100 ppm	2	1	1	2	1
S. coccinea	0.5X | 100 ppm	3	1	2	2	1
S. coccinea	0.5X | 100 ppm	4	2	1	2	2
S. coccinea	0.5X | 100 ppm	5	1	1	1	1
S. coccinea	0.5X | 50 ppm	1	1	1	1	1
S. coccinea	0.5X | 50 ppm	2	1	1	1	1
S. coccinea	0.5X | 50 ppm	3	2	1	2	1
S. coccinea	0.5X | 50 ppm	4	2	1	1	1
S. coccinea	0.5X | 50 ppm	5	1	2	1	1
S. coccinea	1X | 0 ppm	1	1	1	1	1
S. coccinea	1X | 0 ppm	2	0	0	1	1

S. coccinea	1X | 0 ppm	3	1	1	1	1
S. coccinea	1X | 0 ppm	4	1	1	1	1
S. coccinea	1X | 0 ppm	5	1	0	0	1
S. coccinea	1X | 100 ppm	1	2	2	1	1
S. coccinea	1X | 100 ppm	2	1	2	1	1
S. coccinea	1X | 100 ppm	3	1	2	1	1
S. coccinea	1X | 50 ppm	1	2	1	1	1
S. coccinea	1X | 50 ppm	2	0	2	1	2
S. coccinea	1X | 50 ppm	3	1	1	1	1
S. coccinea	1X | 50 ppm	4	1	1	1	1
S. coccinea	1X | 50 ppm	5	0	1	2	2
T. violacea	0.5X | 0 ppm	1	1	1	0	1
T. violacea	0.5X | 0 ppm	2	1	1	1	1
T. violacea	0.5X | 0 ppm	3	0	0	0	0
T. violacea	0.5X | 0 ppm	4	1	2	0	1
T. violacea	0.5X | 100 ppm	1	0	0	0	0
T. violacea	0.5X | 100 ppm	2	0	0	0	0
T. violacea	0.5X | 100 ppm	3	0	1	1	1
T. violacea	0.5X | 50 ppm	1	1	1	1	1
T. violacea	0.5X | 50 ppm	2	2	0	1	1
T. violacea	0.5X | 50 ppm	3	0	1	1	1
T. violacea	1X | 0 ppm	1	0	0	0	0
T. violacea	1X | 0 ppm	2	1	0	0	1
T. violacea	1X | 0 ppm	3	0	0	0	1
T. violacea	1X | 0 ppm	4	0	0	0	0
T. violacea	1X | 100 ppm	1	1	1	0	0
T. violacea	1X | 100 ppm	2	1	0	0	1
T. violacea	1X | 100 ppm	3	1	1	1	1
T. violacea	1X | 50 ppm	1	0	0	1	1
T. violacea	1X | 50 ppm	2	0	1	0	1

Table S3C. Chlorophyll content index values (obtained with a CCM-200 plus chlorophyll content meter) for four leaves (L1-L4) from each individual plant unit within each treatment group (water-resourcing level | initial soil RDX concentration) for eight plant species (*Antirrhinum majus, Dianthus, Hibiscus mocheutos, Plumbago auriculata, Pentas lanceolata, Ruellia caroliniensis, Salvia coccinea, and Tulbaghia violacea*). For each leaf, one to three chlorophyll content index (*CCI*) measurements were made (depending on leaf surface area) using a CCM-200 plus chlorophyll content meter (Opti-Sciences, Inc.; Hudson, NH, USA). Either a single *CCI* value or mean leaf *CCI* was recorded for each leaf. *CCI* is the product of percent light transmittance at 931 nm and the inverse of the percent light transmittance at 653 nm. While not equivalent to actual density of chlorophyll in plant tissues, *CCI* provides a useful metric for comparing chlorophyll content among different samples. "--" denotes a missing datum.

			CCI			
**Plant Species**	**Treatment Group**	**Unit**	**L1**	**L2**	**L3**	**L4**
A. majus	0.5X | 0 ppm	1	4.3	2	2.5	2.8
A. majus	0.5X | 0 ppm	2	3.1	5.5	2.1	6.7
A. majus	0.5X | 0 ppm	3	1.4	1.3	1.8	1.3
A. majus	0.5X | 0 ppm	4	3.4	4.5	2.7	1.2
A. majus	0.5X | 0 ppm	5	4.5	2.4	4.9	1.5
A. majus	0.5X | 100 ppm	1	6.1	2.8	4	1.3
A. majus	0.5X | 100 ppm	2	2.2	2.3	2	2.8
A. majus	0.5X | 100 ppm	3	1.6	1.8	3.5	2.4
A. majus	0.5X | 100 ppm	4	3.9	1.8	2.2	5.5
A. majus	0.5X | 100 ppm	5	3	1.4	2.6	2.1
A. majus	0.5X | 50 ppm	1	1.3	1.3	1.4	1.2
A. majus	0.5X | 50 ppm	2	2.5	3.3	2.9	4.2
A. majus	0.5X | 50 ppm	3	5.2	1.6	1.5	1.7
A. majus	0.5X | 50 ppm	4	3	6.2	1.5	1.6
A. majus	0.5X | 50 ppm	5	9.5	1.7	2.2	4.5
A. majus	1X | 0 ppm	1	2.8	1.6	5.4	3.8
A. majus	1X | 0 ppm	2	6.7	2.2	2.3	4.4
A. majus	1X | 0 ppm	3	2.1	2.4	1.8	3.2
A. majus	1X | 0 ppm	4	4	6.2	4.1	3.8
A. majus	1X | 0 ppm	5	6.5	3.4	1.4	4.3
A. majus	1X | 100 ppm	1	2.2	4.6	5.8	2.7
A. majus	1X | 100 ppm	2	1.2	1.8	1.9	2.7
A. majus	1X | 100 ppm	3	2.4	2.8	2.6	1.9
A. majus	1X | 100 ppm	4	1.7	3.6	1.4	1.5
A. majus	1X | 100 ppm	5	16.7	2.5	3.6	2.7
A. majus	1X | 50 ppm	1	2.1	5.5	2.4	3.4
A. majus	1X | 50 ppm	2	2.8	1.4	2.1	3.5
A. majus	1X | 50 ppm	3	2.4	2.8	2.6	1.9
A. majus	1X | 50 ppm	4	3.7	1.2	1.2	2

A. majus	1X | 50 ppm	5	5.8	8.1	1.9	6.3
Dianthus	0.5X | 0 ppm	1	1.2	1.5	1.1	1.2
Dianthus	0.5X | 0 ppm	2	1	2.2	1.6	6.8
Dianthus	0.5X | 0 ppm	3	1.4	2.7	1.4	1.9
Dianthus	0.5X | 0 ppm	4	3	4.1	13.2	5.5
Dianthus	0.5X | 0 ppm	5	1.4	1.8	7.1	2.1
Dianthus	0.5X | 100 ppm	1	3.1	2.6	4	3
Dianthus	0.5X | 100 ppm	2	3.3	4	4.1	11.2
Dianthus	0.5X | 100 ppm	3	2.8	1.6	6.1	4.2
Dianthus	0.5X | 100 ppm	4	12.6	5.1	4.1	1.8
Dianthus	0.5X | 100 ppm	5	2.5	4	4.4	1.5
Dianthus	0.5X | 50 ppm	5	2.8	2.6	1.5	1
Dianthus	0.5X | 50 ppm	4	1.2	1.4	2.4	4.4
Dianthus	0.5X | 50 ppm	3	1.2	1.5	1.1	1.1
Dianthus	0.5X | 50 ppm	2	1.4	1	1	1.3
Dianthus	0.5X | 50 ppm	1	1	1	1.2	1
Dianthus	1X | 0 ppm	1	10.6	5.8	6.4	1
Dianthus	1X | 0 ppm	2	1.3	12.1	4	6.9
Dianthus	1X | 0 ppm	3	1.2	1.8	5.9	1
Dianthus	1X | 0 ppm	4	2	4.3	5	3.5
Dianthus	1X | 0 ppm	5	1.5	3.3	6	1.6
Dianthus	1X | 100 ppm	1	1.2	5	1.9	3
Dianthus	1X | 100 ppm	2	2.9	7.8	1.2	4.3
Dianthus	1X | 100 ppm	3	4	1.3	2.6	1.1
Dianthus	1X | 100 ppm	4	2.8	1.2	2.3	14.7
Dianthus	1X | 100 ppm	5	1.3	1.4	1.5	7.3
Dianthus	1X | 50 ppm	5	2.3	1.3	1.4	1.6
Dianthus	1X | 50 ppm	4	--	2.1	1.2	1.2
Dianthus	1X | 50 ppm	3	1.1	3.7	1	1.7
Dianthus	1X | 50 ppm	2	1.2	1	1.3	1.3
Dianthus	1X | 50 ppm	1	1	1.3	1.4	1.1
H. mocheutos	0.5X | 0 ppm	1	9.2	11	12.2	15.4
H. mocheutos	0.5X | 0 ppm	2	12	21.8	9.8	17.1
H. mocheutos	0.5X | 0 ppm	3	8.8	18.4	17.6	17.4
H. mocheutos	0.5X | 0 ppm	4	14.9	1.5	14.9	8.4
H. mocheutos	0.5X | 0 ppm	5	13.1	1.2	18.2	11.8
H. mocheutos	0.5X | 100 ppm	1	7.5	2.4	5.4	7.7
H. mocheutos	0.5X | 100 ppm	2	16.1	2.2	16.2	5.4
H. mocheutos	0.5X | 100 ppm	3	6.8	8.6	9.2	13
H. mocheutos	0.5X | 100 ppm	4	9.7	12	12.9	18
H. mocheutos	0.5X | 100 ppm	5	10.6	9.4	12.5	12.3
H. mocheutos	0.5X | 50 ppm	1	10.8	12.1	12.1	10.9
H. mocheutos	0.5X | 50 ppm	2	14	13.6	3.7	13.6
H. mocheutos	0.5X | 50 ppm	3	1.1	1	3.7	1.6
H. mocheutos	0.5X | 50 ppm	4	8.9	7.8	9.7	9.6
H. mocheutos	0.5X | 50 ppm	5	9.6	9.5	11.6	9.3

H. mocheutos	1X | 0 ppm	1	7.6	9.1	7.3	11.8
H. mocheutos	1X | 0 ppm	2	20	20.9	19.6	18.6
H. mocheutos	1X | 0 ppm	3	12.6	7.1	7.1	5.7
H. mocheutos	1X | 0 ppm	4	4	5.5	10.9	6.9
H. mocheutos	1X | 100 ppm	1	11.7	6.3	5.5	9.9
H. mocheutos	1X | 100 ppm	2	2	1.2	1.6	3
H. mocheutos	1X | 100 ppm	3	6.1	5.1	8.2	9.9
H. mocheutos	1X | 100 ppm	4	13.5	13.2	9.4	12.9
H. mocheutos	1X | 100 ppm	5	16.4	19	10	11.1
H. mocheutos	1X | 50 ppm	1	2.3	7	5.9	6.8
H. mocheutos	1X | 50 ppm	2	13	13.3	17.6	15.6
H. mocheutos	1X | 50 ppm	3	14.2	19.2	5.6	14.7
H. mocheutos	1X | 50 ppm	4	15.9	14.9	7.9	5.6
H. mocheutos	1X | 50 ppm	5	24.3	8.5	6	19.7
P. auriculata	0.5X | 0 ppm	1	7.3	10.2	5.9	9.1
P. auriculata	0.5X | 0 ppm	2	17.3	12.3	11.1	10.2
P. auriculata	0.5X | 100 ppm	1	7.3	7	7.6	2.3
P. auriculata	0.5X | 100 ppm	2	7	11.4	7.9	7.1
P. auriculata	0.5X | 100 ppm	3	7.2	4	9.5	5.5
P. auriculata	0.5X | 50 ppm	1	8.7	7.1	11	7.7
P. auriculata	0.5X | 50 ppm	2	5.6	10.1	8.1	13.1
P. auriculata	0.5X | 50 ppm	3	9.4	6.2	6.9	6.5
P. auriculata	1X | 0 ppm	1	13.3	12.5	5.7	12.1
P. auriculata	1X | 0 ppm	2	5.1	12.3	5.6	5.2
P. auriculata	1X | 100 ppm	1	7.6	5.4	4.8	4.5
P. auriculata	1X | 100 ppm	2	5.4	9.5	8.2	5.2
P. auriculata	1X | 100 ppm	3	7.6	8.9	6	5.2
P. auriculata	1X | 50 ppm	1	7.3	4.2	5.7	3.8
P. auriculata	1X | 50 ppm	2	3.1	1.9	3.3	6.2
P. auriculata	1X | 50 ppm	3	4	3.9	5.5	4.6
P. lanceolata	0.5X | 0 ppm	1	3.2	32.3	32.3	2.4
P. lanceolata	0.5X | 0 ppm	2	40.4	37.6	30.3	45.4
P. lanceolata	0.5X | 0 ppm	3	17.9	21.5	11.2	26
P. lanceolata	0.5X | 0 ppm	4	14.8	9.1	2	9.2
P. lanceolata	0.5X | 0 ppm	5	18.9	17.2	26.5	16.4
P. lanceolata	0.5X | 100 ppm	1	21.3	23.6	24.3	22.3
P. lanceolata	0.5X | 100 ppm	2	24.9	25.9	26.3	17.1
P. lanceolata	0.5X | 100 ppm	3	44	31.3	25.1	37.9
P. lanceolata	0.5X | 100 ppm	4	47.6	28.1	28.1	23.9
P. lanceolata	0.5X | 100 ppm	5	10.8	14	11.3	13.2
P. lanceolata	0.5X | 50 ppm	1	18.6	28.2	28.1	31.8
P. lanceolata	0.5X | 50 ppm	2	4.3	12.5	5	8.7
P. lanceolata	0.5X | 50 ppm	3	13.9	23	17.7	19
P. lanceolata	0.5X | 50 ppm	4	22	22.7	40	46.5
P. lanceolata	0.5X | 50 ppm	5	23	32.7	15	35
P. lanceolata	1X | 0 ppm	1	21.3	15.1	26.9	11.6

P. lanceolata	1X | 0 ppm	2	27	15.5	15.4	26.3
P. lanceolata	1X | 0 ppm	3	23.2	34.9	45.1	24.2
P. lanceolata	1X | 0 ppm	4	33.2	30.4	27.9	23.4
P. lanceolata	1X | 0 ppm	5	17.4	27	7.8	6.8
P. lanceolata	1X | 100 ppm	1	7.3	7.7	19.5	4.7
P. lanceolata	1X | 100 ppm	2	2.1	7.1	40.6	17.6
P. lanceolata	1X | 100 ppm	3	12.1	26.2	22.6	12.3
P. lanceolata	1X | 100 ppm	4	11.8	15.2	19.1	14.9
P. lanceolata	1X | 100 ppm	5	31	23.3	16.5	5.7
P. lanceolata	1X | 50 ppm	1	7.2	7.6	20.1	20.7
P. lanceolata	1X | 50 ppm	2	20.3	17	14.8	16.5
P. lanceolata	1X | 50 ppm	3	5.8	14.9	11.9	15.1
P. lanceolata	1X | 50 ppm	4	15.1	25.9	21	9.9
P. lanceolata	1X | 50 ppm	5	25.7	12.7	18.8	25.6
R. caroliniensis	0.5X | 0 ppm	1	22.2	20.8	12.4	20.7
R. caroliniensis	0.5X | 0 ppm	2	9.5	11.9	17.4	14.4
R. caroliniensis	0.5X | 0 ppm	3	7.9	12.2	10.5	22.5
R. caroliniensis	0.5X | 0 ppm	4	18.8	27.4	7.4	25
R. caroliniensis	0.5X | 100 ppm	1	10.5	9.8	8.4	14.5
R. caroliniensis	0.5X | 100 ppm	2	6	13.7	12	11.1
R. caroliniensis	0.5X | 100 ppm	3	7.4	1.4	5.4	1.7
R. caroliniensis	0.5X | 100 ppm	4	45.1	8.4	5.3	4.6
R. caroliniensis	0.5X | 50 ppm	1	10.6	18.5	16.1	10
R. caroliniensis	0.5X | 50 ppm	2	4.1	10.5	15.9	2.2
R. caroliniensis	0.5X | 50 ppm	3	15.5	25.2	2.3	2
R. caroliniensis	0.5X | 50 ppm	4	29.1	51.4	12.8	2
R. caroliniensis	1X | 0 ppm	1	13.7	27.6	9.7	20.2
R. caroliniensis	1X | 0 ppm	2	11.9	45.1	10.5	42.8
R. caroliniensis	1X | 0 ppm	3	16.3	2.8	8	9.1
R. caroliniensis	1X | 100 ppm	1	8.6	18.2	40.8	29.6
R. caroliniensis	1X | 100 ppm	2	11.8	6	20.8	3
R. caroliniensis	1X | 100 ppm	3	2.1	1.5	7.9	1.7
R. caroliniensis	1X | 100 ppm	4	1.9	6.3	22.3	3.7
R. caroliniensis	1X | 50 ppm	1	20.9	14	3.9	5.3
R. caroliniensis	1X | 50 ppm	2	15.8	8.8	3.9	12.7
R. caroliniensis	1X | 50 ppm	3	4.6	9.5	31.6	4.8
R. caroliniensis	1X | 50 ppm	4	5	33.5	19.7	30.3
S. coccinea	0.5X | 0 ppm	1	15.2	9.2	17.8	22.6
S. coccinea	0.5X | 0 ppm	2	13.8	12.5	11.3	10.2
S. coccinea	0.5X | 0 ppm	3	9.3	13.1	9.2	9.8
S. coccinea	0.5X | 0 ppm	4	7.3	9	11.2	6.7
S. coccinea	0.5X | 0 ppm	5	11.2	9.9	10.2	11.1
S. coccinea	0.5X | 100 ppm	1	11.6	15.9	12.4	6
S. coccinea	0.5X | 100 ppm	2	10.1	17.2	14.1	16.1
S. coccinea	0.5X | 100 ppm	3	9.8	9.2	8.8	13.6
S. coccinea	0.5X | 100 ppm	4	9.8	12.1	11	8

S. coccinea	0.5X | 100 ppm	5	17.7	15	9.9	11.9
S. coccinea	0.5X | 50 ppm	1	14.6	16.6	9.6	7.8
S. coccinea	0.5X | 50 ppm	2	16.5	12.8	12.5	10.4
S. coccinea	0.5X | 50 ppm	3	11.4	10.2	9.3	9.3
S. coccinea	0.5X | 50 ppm	4	9.3	12.4	8.6	17.6
S. coccinea	0.5X | 50 ppm	5	10.6	9.6	10.3	9.2
S. coccinea	1X | 0 ppm	1	13.9	10.5	10.2	10.6
S. coccinea	1X | 0 ppm	2	12.3	17.5	13.2	14
S. coccinea	1X | 0 ppm	3	10.1	8.3	11.5	7.6
S. coccinea	1X | 0 ppm	4	8.5	13.1	2.6	9.4
S. coccinea	1X | 0 ppm	5	16	11	17.3	14.4
S. coccinea	1X | 100 ppm	1	8.4	7.1	9.5	9.7
S. coccinea	1X | 100 ppm	2	13.4	8.4	8.5	5.4
S. coccinea	1X | 100 ppm	3	5.7	6.1	5.1	9.2
S. coccinea	1X | 100 ppm	4	16.5	13.2	13.2	10.5
S. coccinea	1X | 100 ppm	5	8.2	9.5	12.7	13.1
S. coccinea	1X | 50 ppm	1	7.1	12.8	9.1	8.3
S. coccinea	1X | 50 ppm	2	8.5	5.8	10.2	12.3
S. coccinea	1X | 50 ppm	3	10.5	14.4	8.5	10.1
S. coccinea	1X | 50 ppm	4	14.7	10.5	11.9	9.5
S. coccinea	1X | 50 ppm	5	10.7	19.8	10.5	14.2
T. violacea	0.5X | 0 ppm	1	10.3	3.3	9.8	5
T. violacea	0.5X | 0 ppm	2	2.3	3.3	9.2	5.7
T. violacea	0.5X | 0 ppm	3	2.8	1.8	1.7	3.7
T. violacea	0.5X | 0 ppm	4	7.3	3.5	2.3	5.1
T. violacea	0.5X | 100 ppm	1	10.8	3.4	4.9	2.7
T. violacea	0.5X | 100 ppm	2	1.8	1.7	2.1	1.3
T. violacea	0.5X | 100 ppm	3	7.4	3.7	3.5	5.4
T. violacea	0.5X | 50 ppm	1	4	13	4.2	15
T. violacea	0.5X | 50 ppm	2	3.1	1.7	4.4	2.7
T. violacea	0.5X | 50 ppm	3	2.1	1.7	3.4	1.4
T. violacea	1X | 0 ppm	1	2.4	2.3	1.4	1.7
T. violacea	1X | 0 ppm	2	6.7	3.3	2.2	3.1
T. violacea	1X | 0 ppm	3	3.9	5.2	3.1	14.5
T. violacea	1X | 0 ppm	4	2	1.8	3.3	2.9
T. violacea	1X | 100 ppm	1	2.2	1.7	2.4	4.1
T. violacea	1X | 100 ppm	2	2.1	2.1	1.6	1.4
T. violacea	1X | 100 ppm	3	7.9	2.6	4.1	6.9
T. violacea	1X | 50 ppm	1	5.1	17.7	4.3	4.9
T. violacea	1X | 50 ppm	2	7.2	10.4	4.9	--
