# Supplementary material for: Multiple stressors in multiple species: Effects of different RDX soil concentrations and differential water-resourcing on RDX fate, plant health, and plant survival
Source: PLoS One. 2020 Aug 14;15(8):e0234166. doi: 10.1371/journal.pone.0234166 (PMC7428167; doi:10.1371/journal.pone.0234166)
Supplement: S4 Table — A and B. Soil and shoot RDX concentration data from outdoor plot trial. Tables of soil (Table A) and shoot (Table B) RDX concentrations (“soil_rdx,” “shoot_rdx,” respectively) for individual plants within each treatment group (“treatment”) and within each of three species (“plant_species”; Table 1). Treatment groups were based on different initial soil concentrations of RDX (“rdx”) and water-resourcing (“water”). (PDF) [file pone.0234166.s005.pdf]

S4
Soil and Shoot RDX Concentrations from the Outdoor Plot Trial

Table S4A. Soil RDX concentrations (ppm) for three individual plants per species within each treatment group (water-resourcing level | initial soil RDX concentration) for three plant species (*Conradina canescens, Pentas lanceolata,* and *Ruellia caroliniensis*).

Plant species	Treatment Group	Soil RDX (ppm)
COCA	1X	100 ppm	6.31
COCA	1X	100 ppm	5.69
COCA	1X	100 ppm	6.29
PELA	1X	100 ppm	9.61
PELA	1X	100 ppm	14.55
PELA	1X	100 ppm	14.80
RUCA	1X	100 ppm	7.74
RUCA	1X	100 ppm	21.21
RUCA	1X	100 ppm	14.73
COCA	0.5X	100 ppm	10.40
COCA	0.5X	100 ppm	11.12
COCA	0.5X	100 ppm	16.85
PELA	0.5X	100 ppm	19.09
PELA	0.5X	100 ppm	23.18
PELA	0.5X	100 ppm	13.62
RUCA	0.5X	100 ppm	20.78
RUCA	0.5X	100 ppm	20.08
RUCA	0.5X	100 ppm	19.89

Table S4B. Shoot RDX concentrations (ppm) for three individual plants per species within each treatment group (water-resourcing level | initial soil RDX concentration) for three plant species (*Conradina canescens, Pentas lanceolata,* and *Ruellia caroliniensis*).

Plant species	Treatment Group	Shoot RDX (ppm)
COCA	1X	100 ppm	40.04
COCA	1X	100 ppm	268.97
COCA	1X	100 ppm	122.59
RUCA	1X	100 ppm	117.71
RUCA	1X	100 ppm	13.7
RUCA	1X	100 ppm	161.67
PELA	1X	100 ppm	62.84
PELA	1X	100 ppm	25.11
PELA	1X	100 ppm	34.04
COCA	0.5X	100 ppm	224.63

COCA	0.5X | 100 ppm	404.51
COCA	0.5X | 100 ppm	210.63
RUCA	0.5X | 100 ppm	123.88
RUCA	0.5X | 100 ppm	166.82
RUCA	0.5X | 100 ppm	129.65
PELA	0.5X | 100 ppm	227.6
PELA	0.5X | 100 ppm	20.21
PELA	0.5X | 100 ppm	111.66
